# Supplementary material for: Cytochalasans from the Endophytic Fungus Aspergillus sp. LE2: Their Structures, Antibacterial and NO Production Inhibitory Activities
Source: J Fungi (Basel). 2023 Mar 19;9(3):374. doi: 10.3390/jof9030374 (PMC10051723; doi:10.3390/jof9030374)
Supplement: Supplementary file 1 [file jof-09-00374-s001.zip › Supporting Information.pdf]

Supporting Information for

Cytochalasans from the Endophytic Fungus *Aspergillus* sp. LE2: Their Structures, Antibacterial and NO Production Inhibitory Activities

Yong-Chao Li [1,3,*], Jing Yang [1,3], Yuan-Zeng Zhao [1,3,], Qin-Di Ma [1,3,], Ben-Guo Liu [2,3], and Jun-Liang Sun [2,3]

[1] School of Life Science and Technology, Henan Institute of Science and Technology, Xinxiang 453003, People's Republic of China

[2] School of Food Science, Henan Institute of Science and Technology, Xinxiang 453003, People's Republic of China

[3] Henan International Joint Laboratory of Plant Genetic Improvement and Soil Remediation, Xinxiang 453003, People's Republic of China

*Correspondence: histlyc@163.com (Y. C. L.)

Contents

Section S1. NMR and MS spectra for Compounds 1–6.

Figure S1-S7. NMR and mass spectra of aspergicytochalasin A (**1**).

Figure S8-S14. NMR and mass spectra of aspergicytochalasin B (**2**).

Figure S15-S21. NMR and mass spectra of aspergicytochalasin C (**3**).

Figure S22-S28. NMR and mass spectra of aspergicytochalasin D (**4**).

Figure S29-S35. NMR and mass spectra of aspergicytochalasin E (**5**).

Figure S36-S42. NMR and mass spectra of aspergicytochalasin F (**6**).

Section S2. X-Ray Crystallographic Data for aspergicytochalasin A (1).

Section S1. NMR and MS spectra for Compounds 1–6.

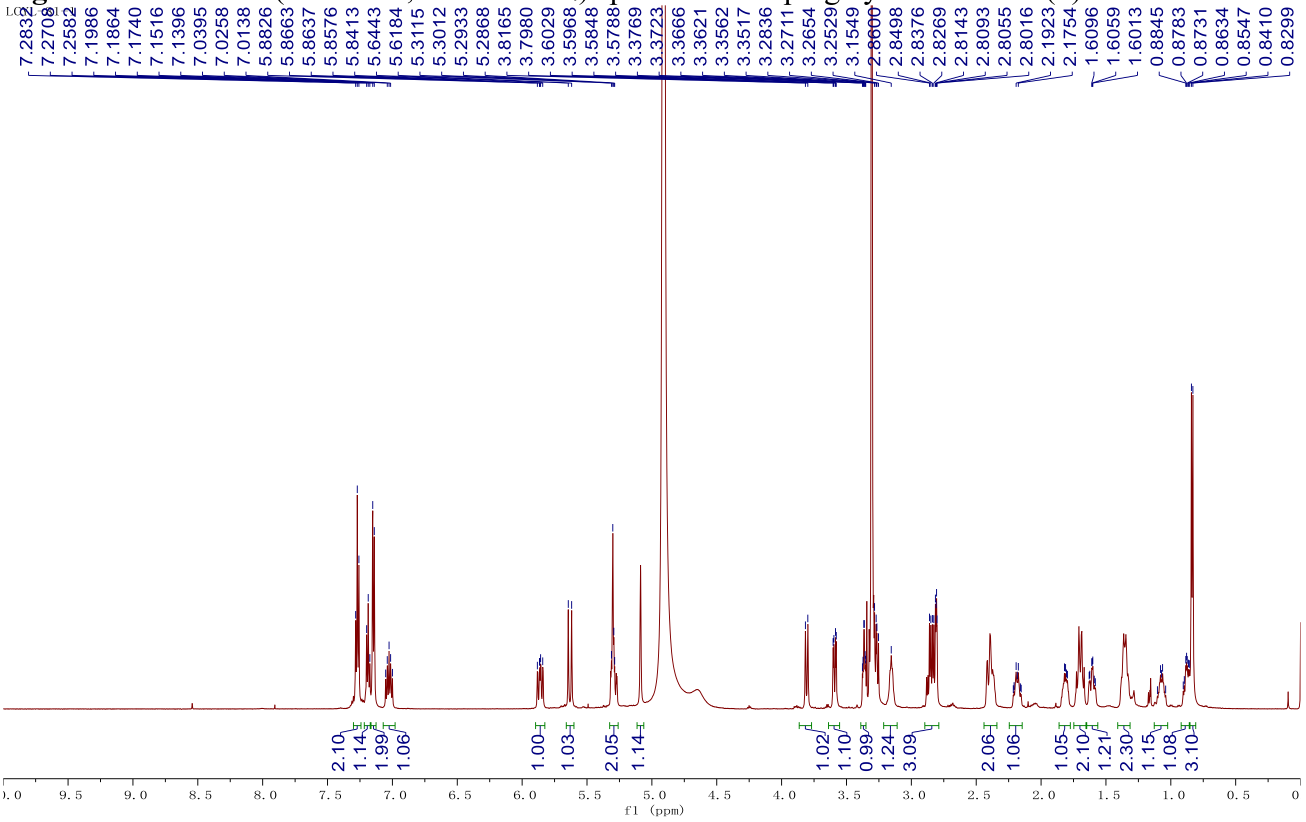

Figure S1. ^{1}H NMR (600 MHz, methanol-d_4) spectrum of aspergicytochalasin A (**1**)

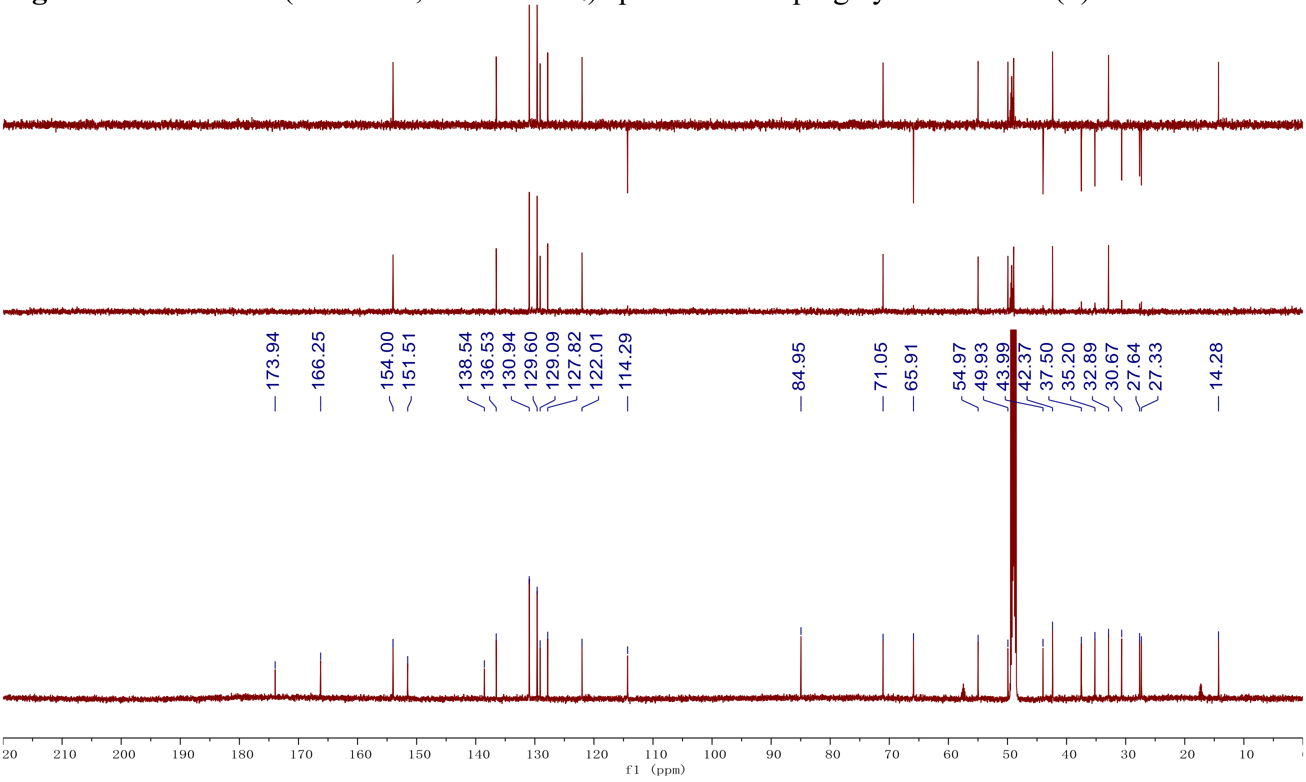

Figure S2. ^{13}C NMR (150 MHz, methanol-d_4) spectrum of aspergicytochalasin A (**1**)

Figure S3. HSQC spectrum of aspergicytochalasin A (**1**)

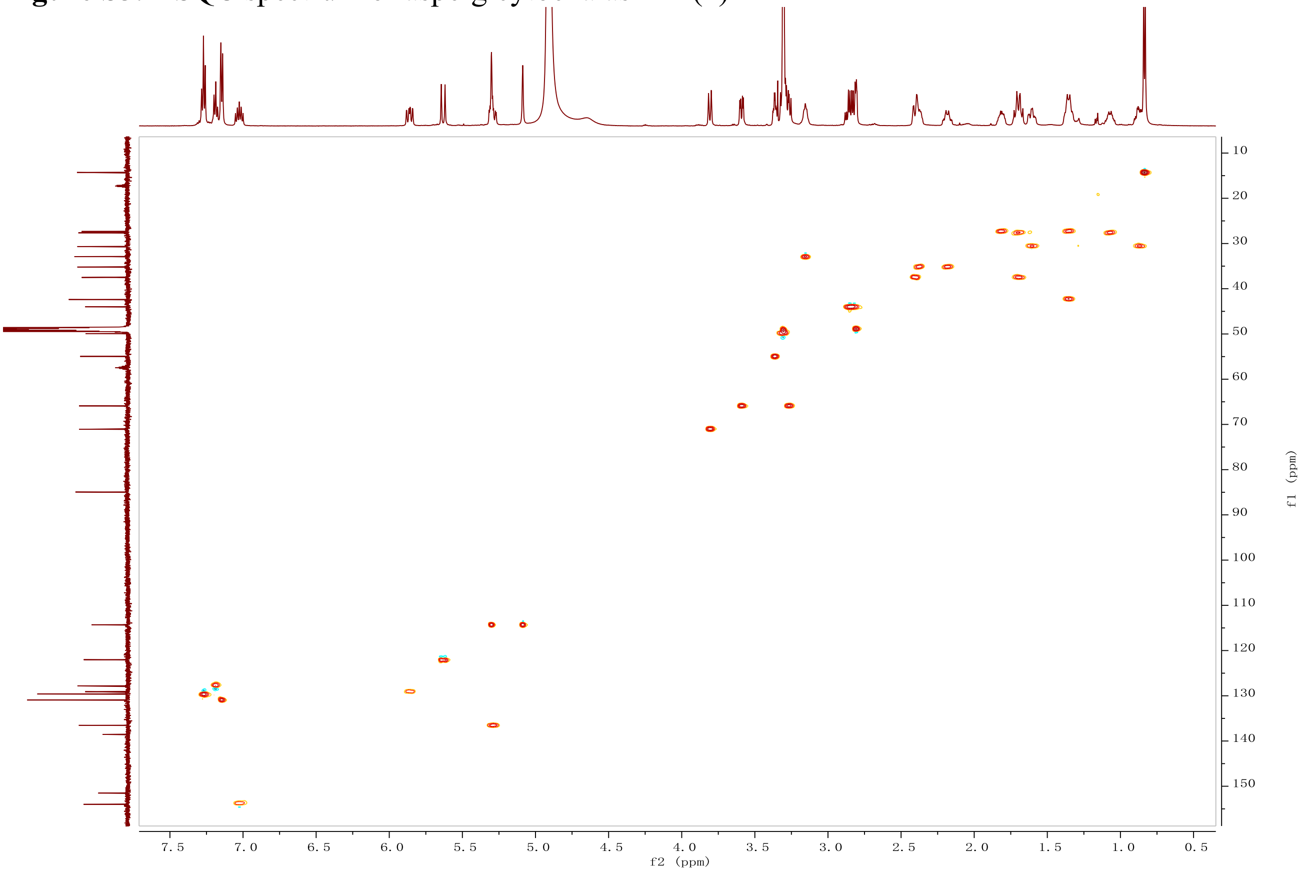

Figure S4. HMBC spectrum of aspergicytochalasin A (**1**)

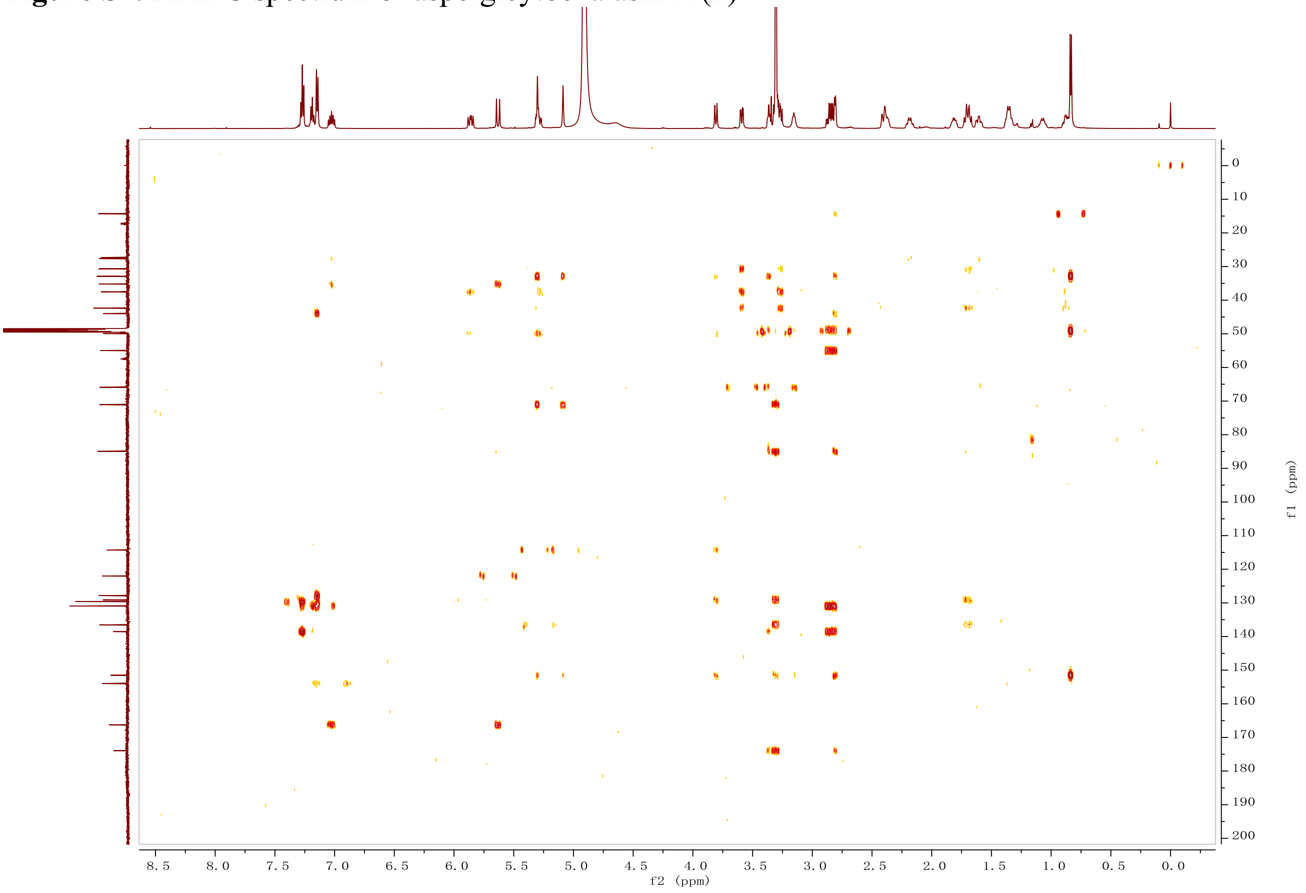

Figure S5. ^{1}H-^{1}H COSY spectrum of aspergicytochalasin A (**1**)

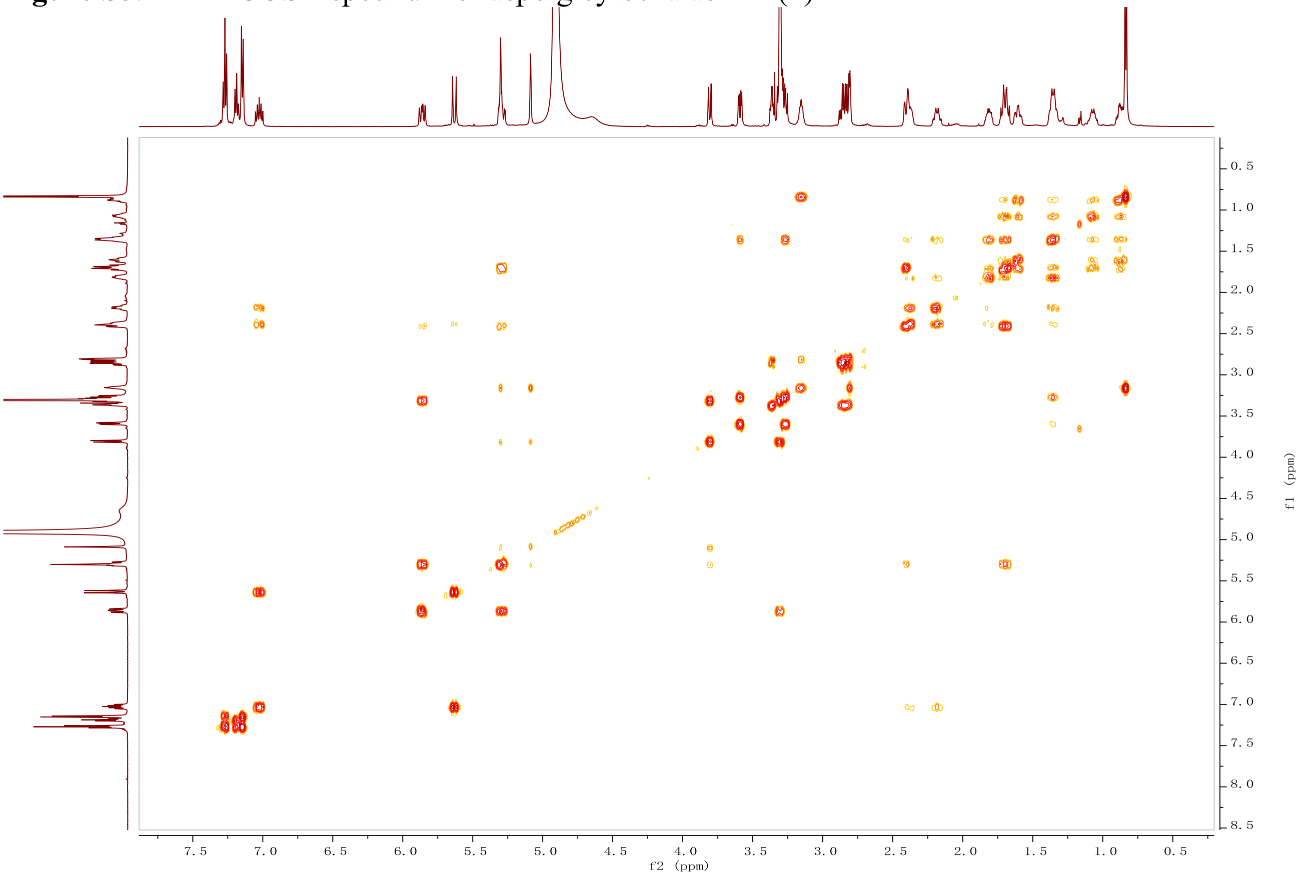

Figure S6. ROESY spectrum of aspergicytochalasin A (**1**)

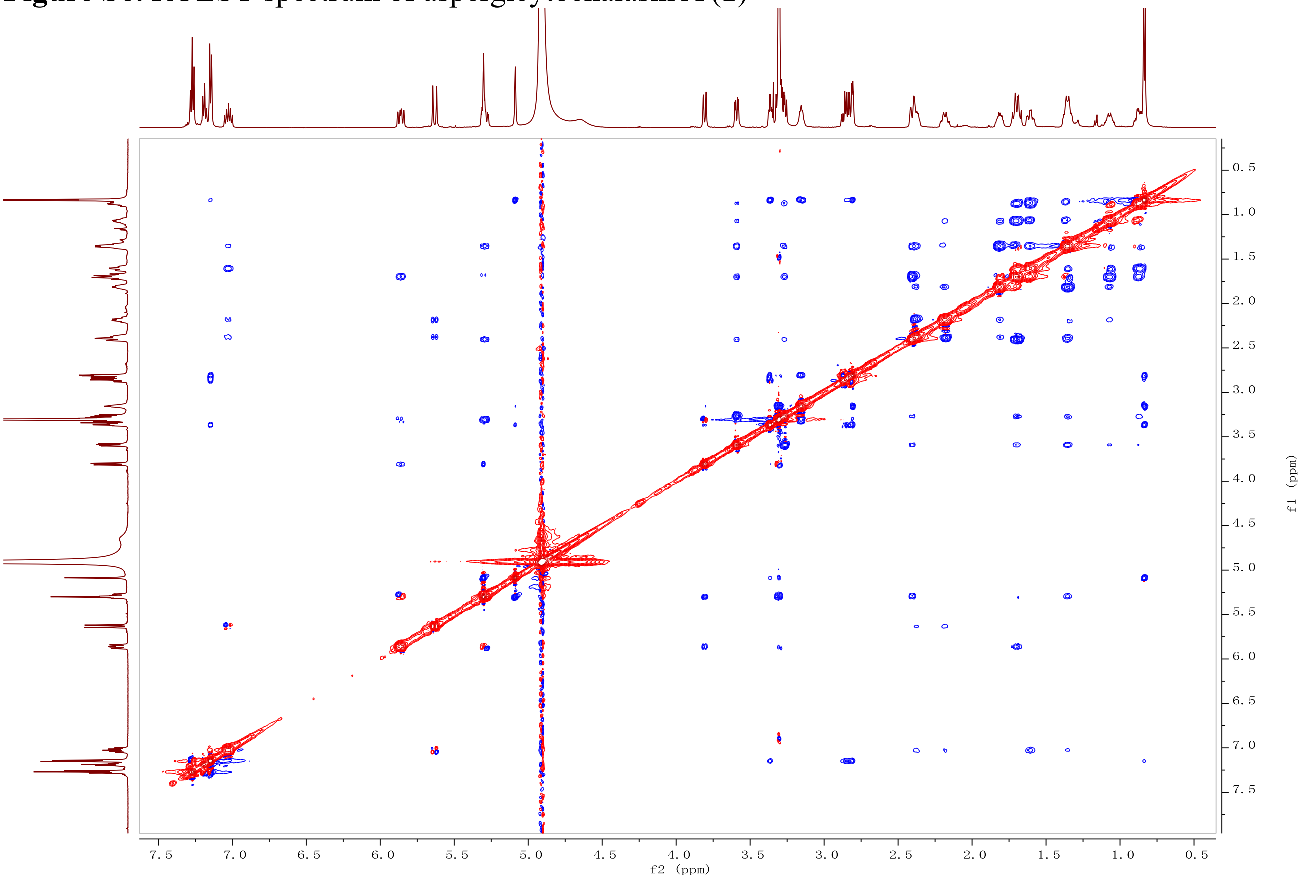

Figure S7. HRESIMS of aspergicytochalasin A (**1**)

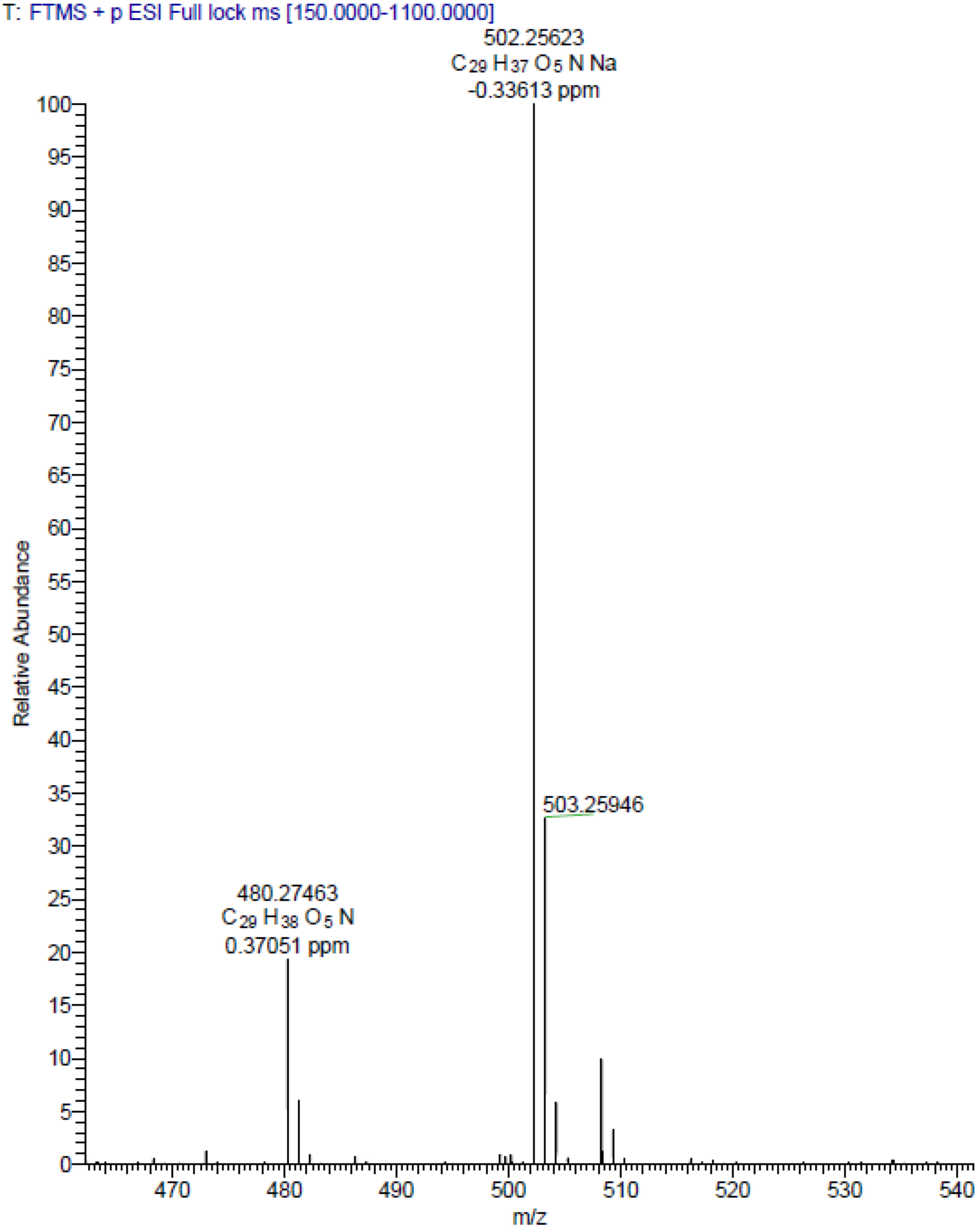

Figure S8. ^{1}H NMR (600 MHz, methanol-d_4) spectrum of aspergicytochalasin B (**2**)

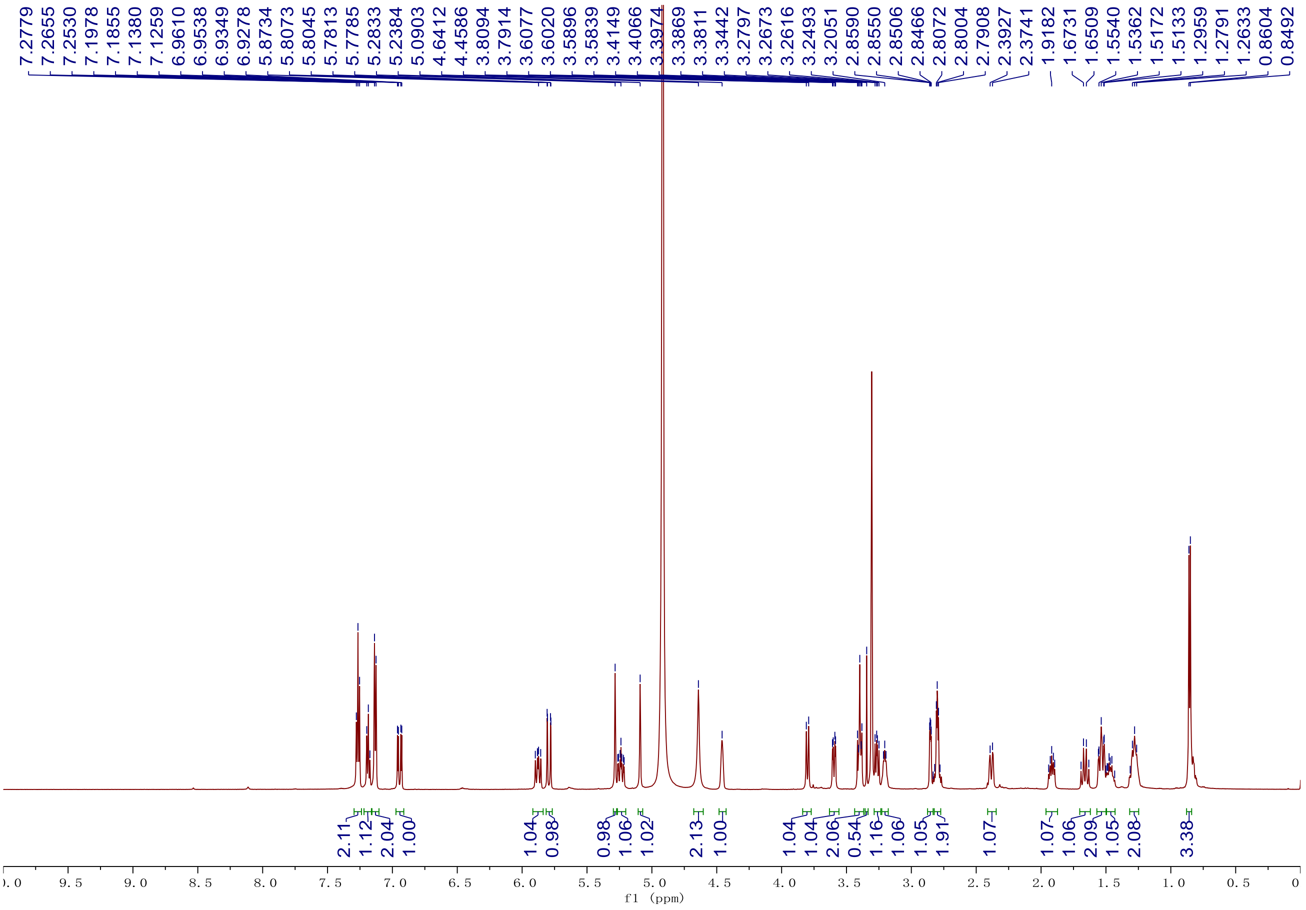

Figure S9. ^{13}C NMR (150 MHz, methanol-d_4) spectrum of aspergicytochalasin B (**2**)

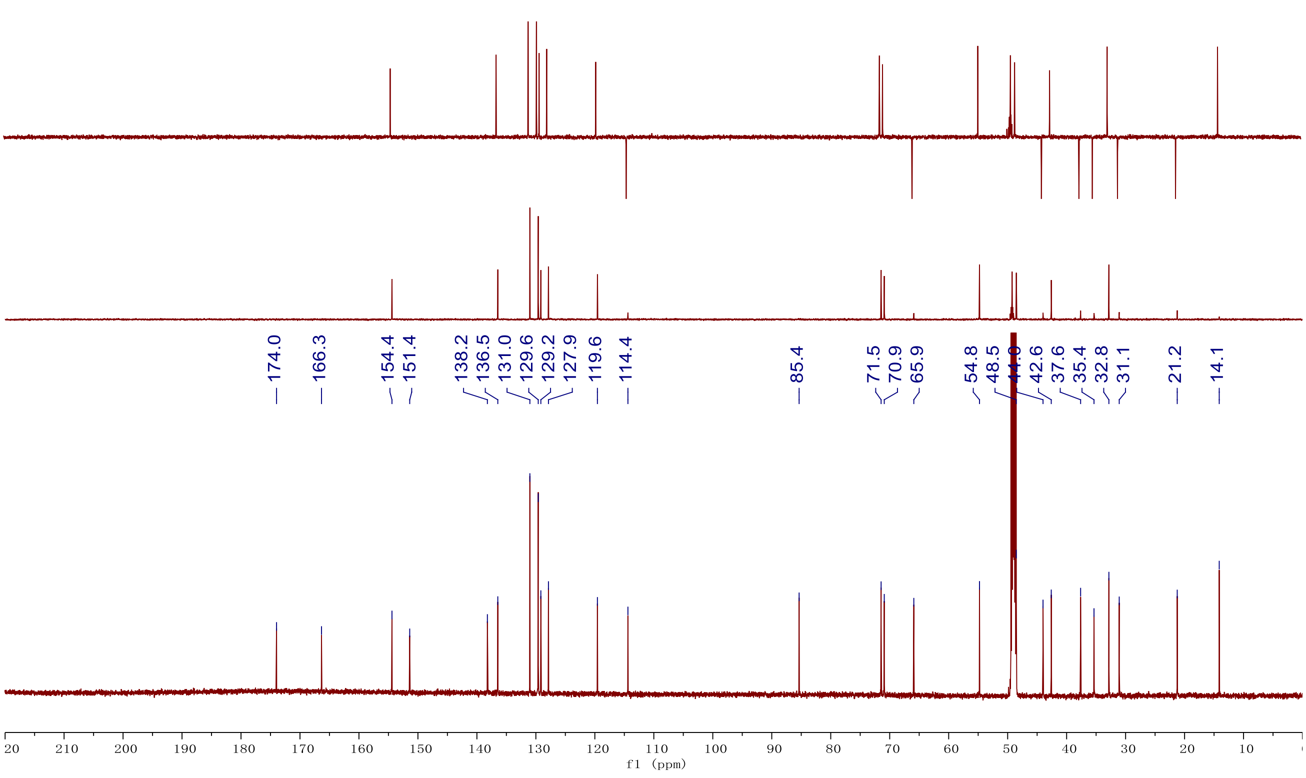

Figure S10. HSQC spectrum of aspergicytochalasin B (**2**)

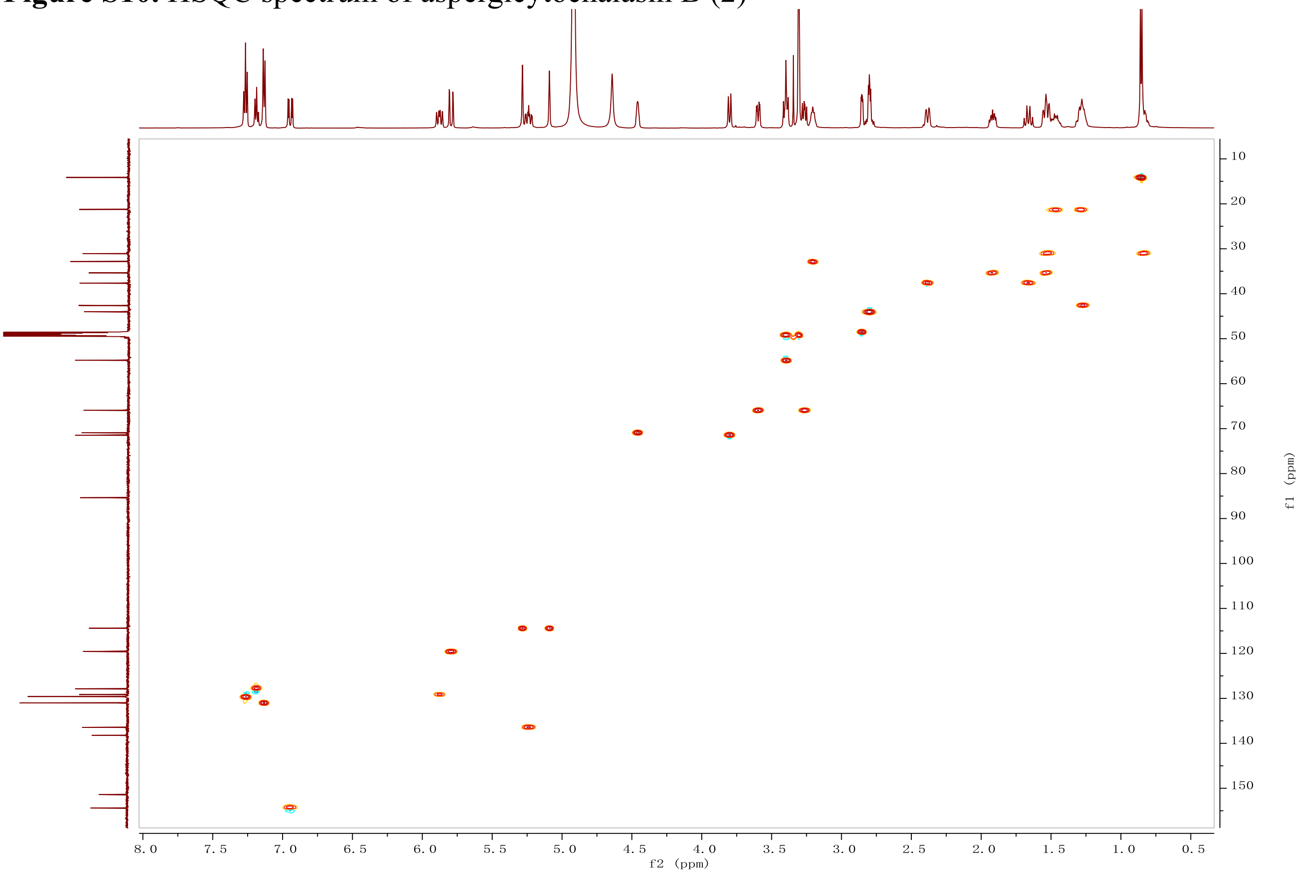

Figure S11. HMBC spectrum of aspergicytochalasin B (**2**)

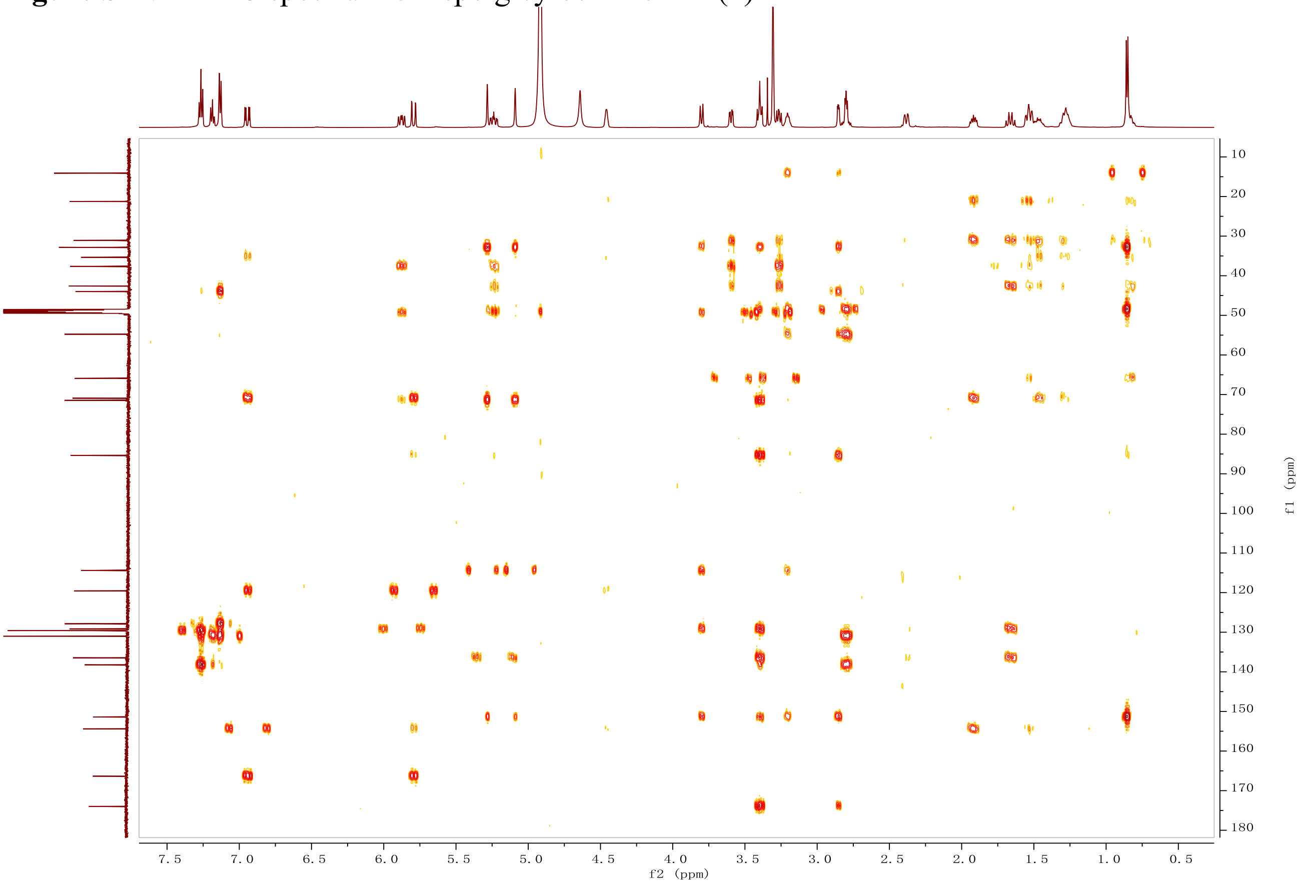

Figure S12. ^{1}H-^{1}H COSY spectrum of aspergicytochalasin B (**2**)

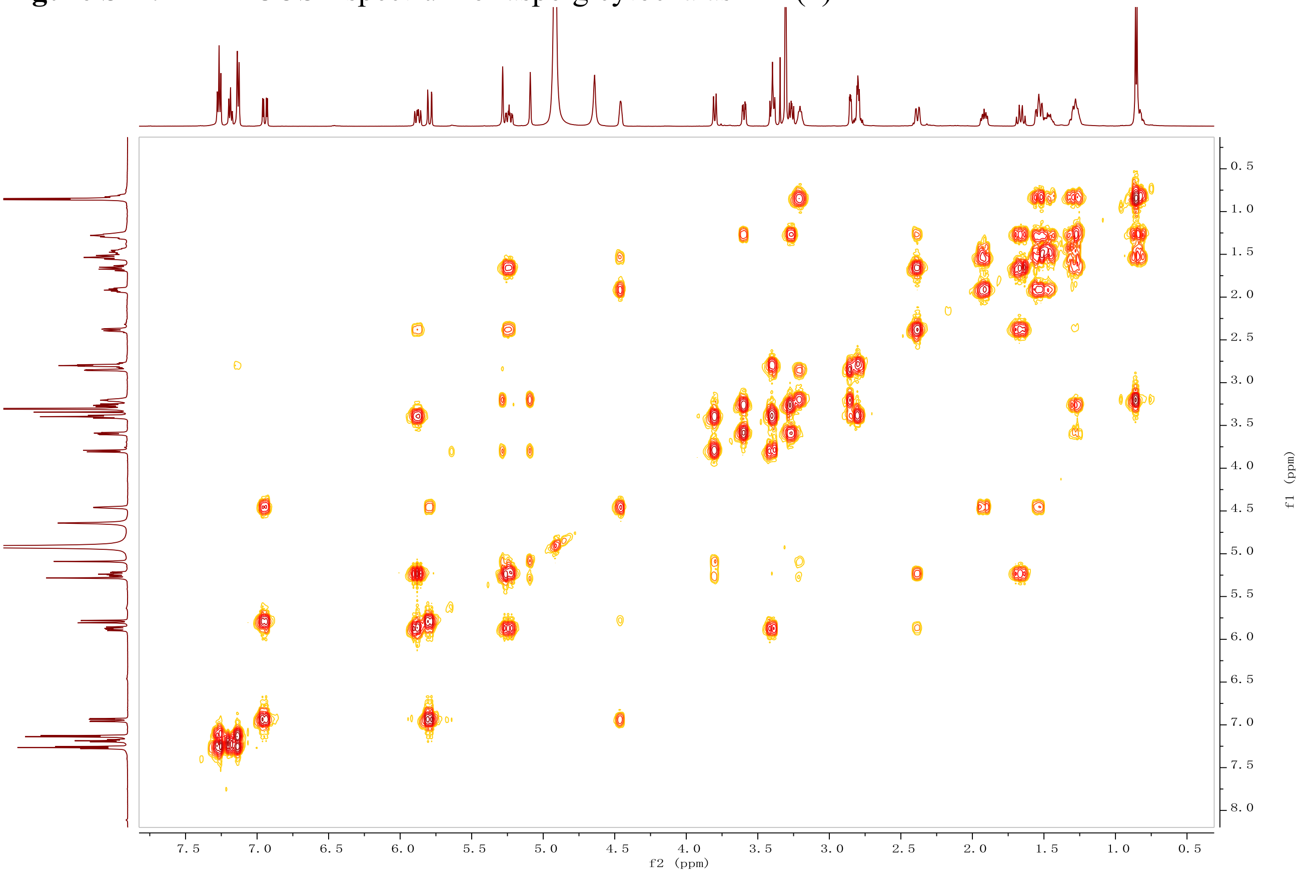

Figure S13. ROESY spectrum of aspergicytochalasin B (**2**)

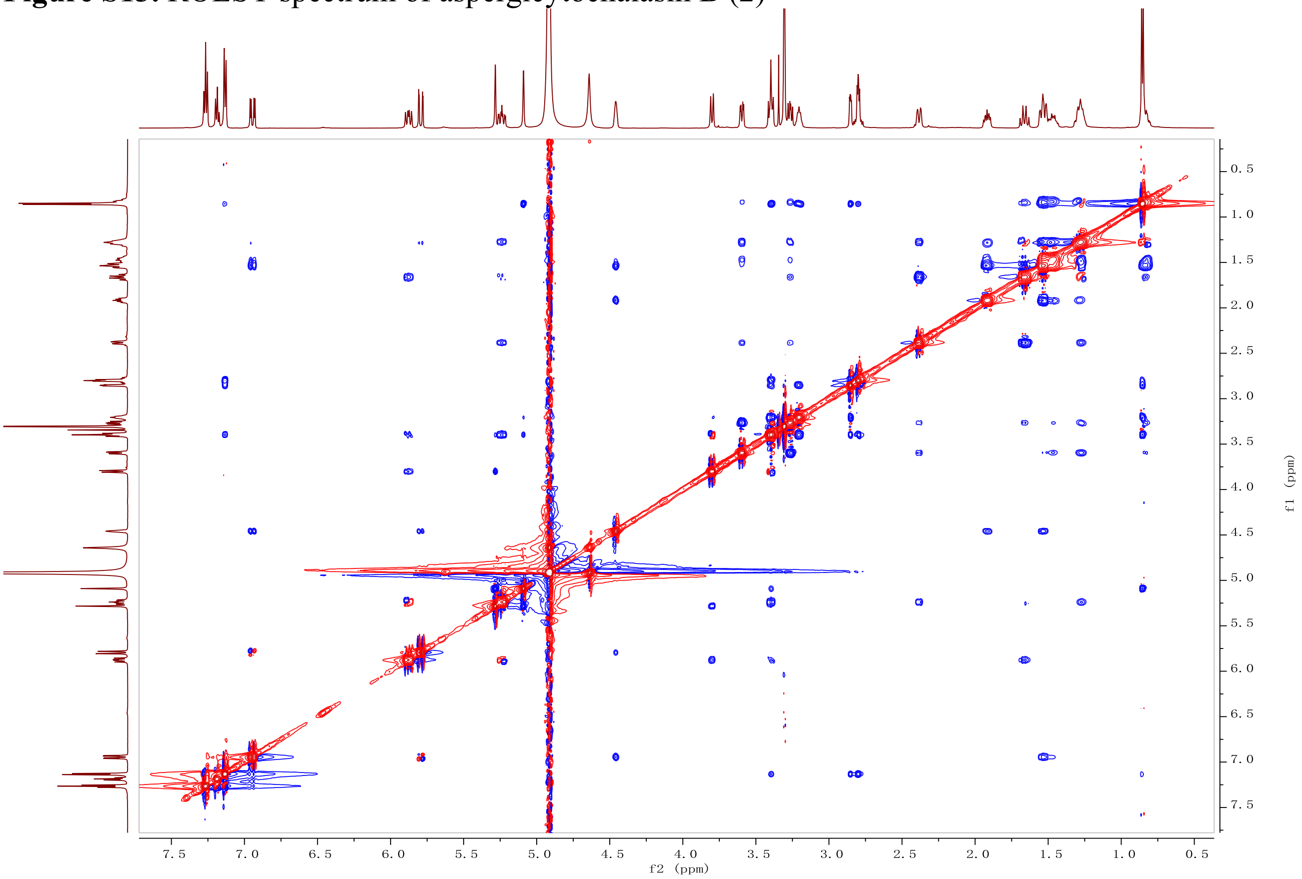

Figure S14. HRESIMS of aspergicytochalasin B (**2**)

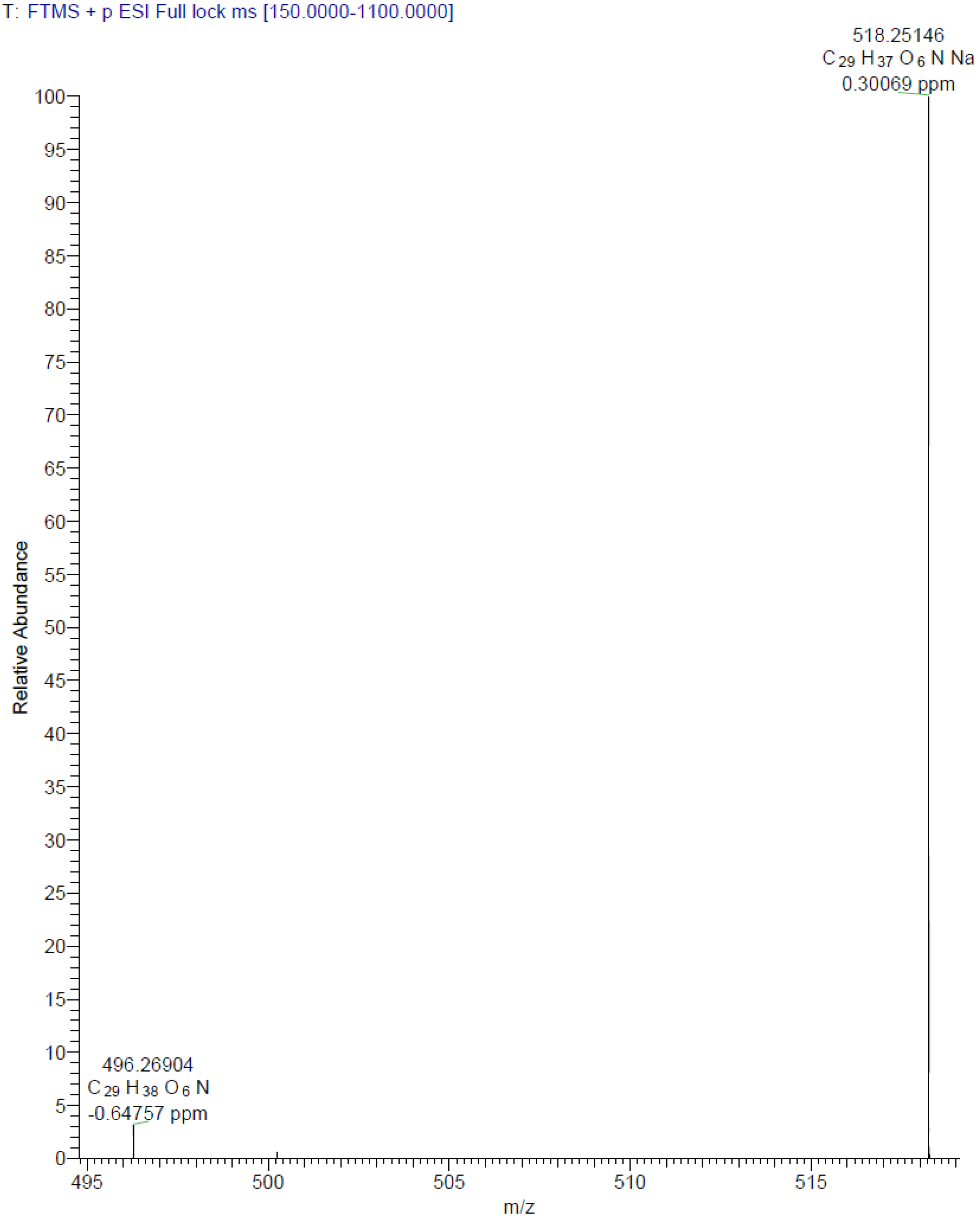

Figure S15. ^{1}H NMR (600 MHz, methanol-d_4) spectrum of aspergicytochalasin C (**3**)

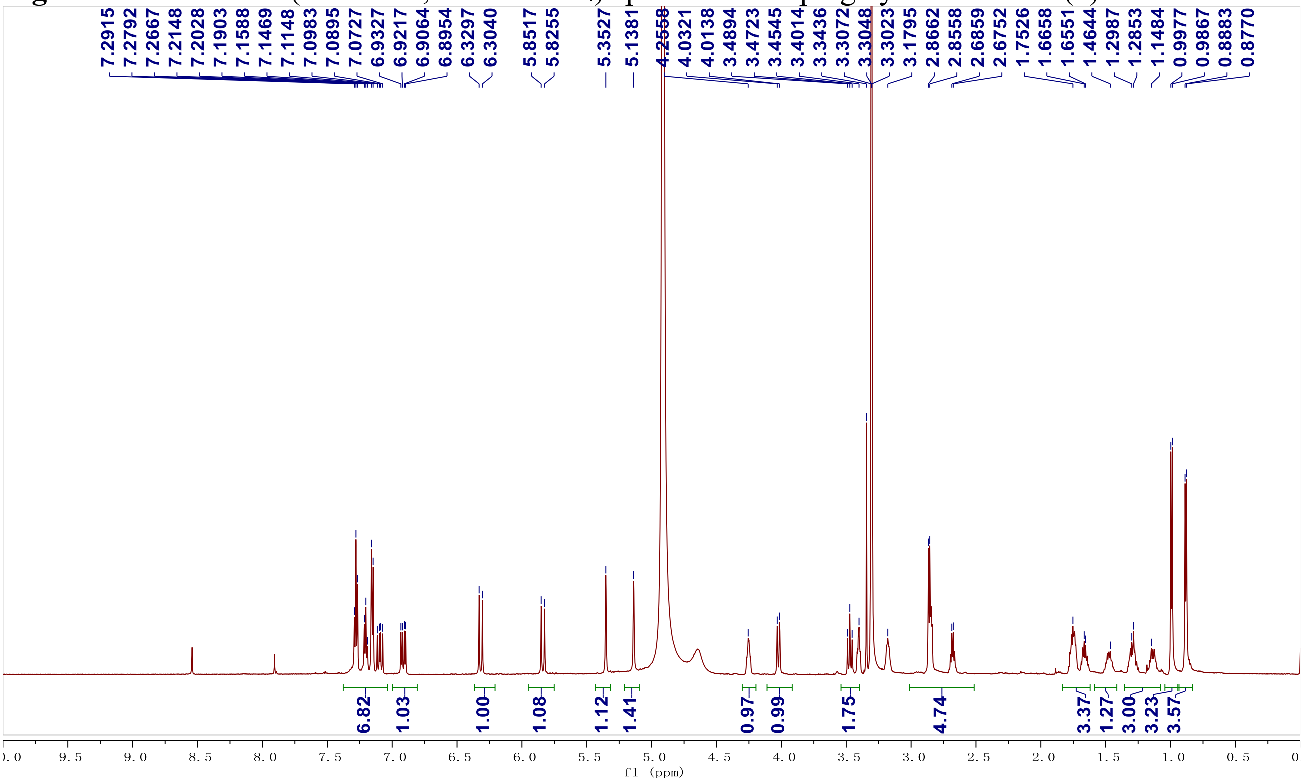

Figure S16. ^{13}C NMR (150 MHz, methanol-d_4) spectrum of aspergicytochalasin C (**3**)

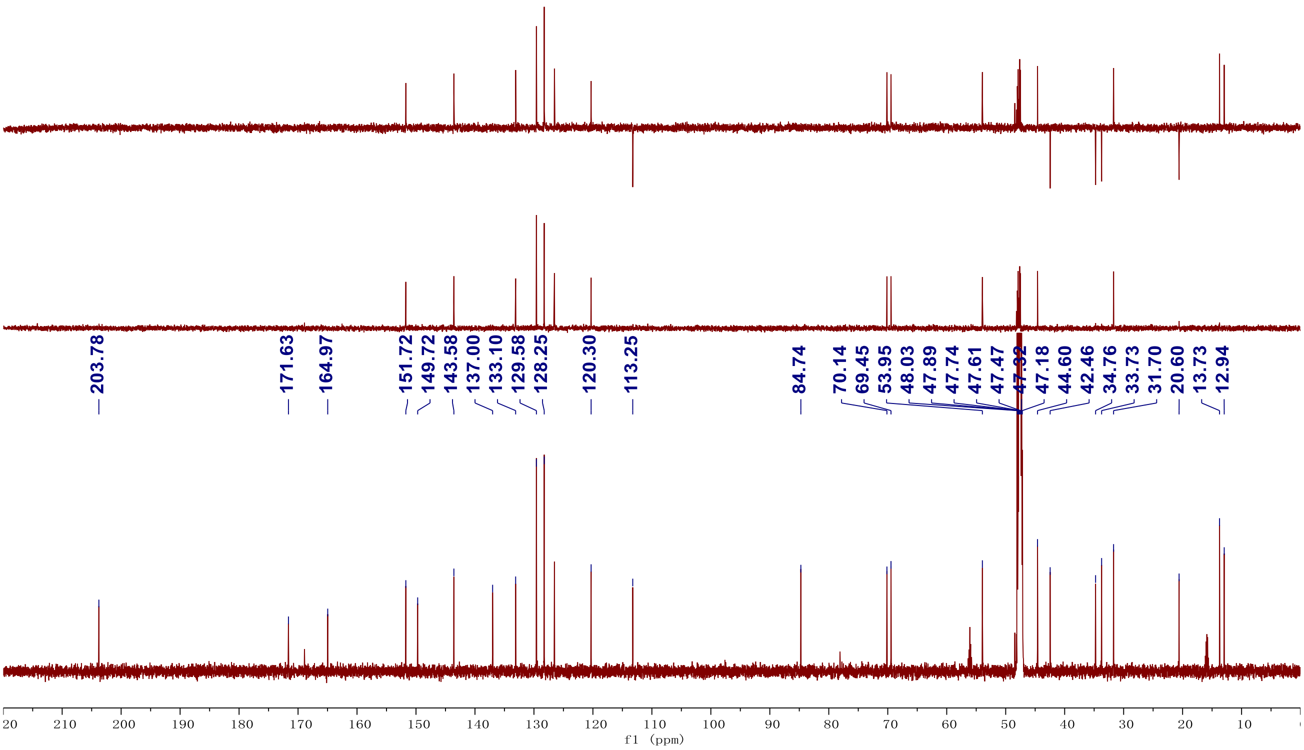

Figure S17. HSQC spectrum of aspergicytochalasin C (**3**)

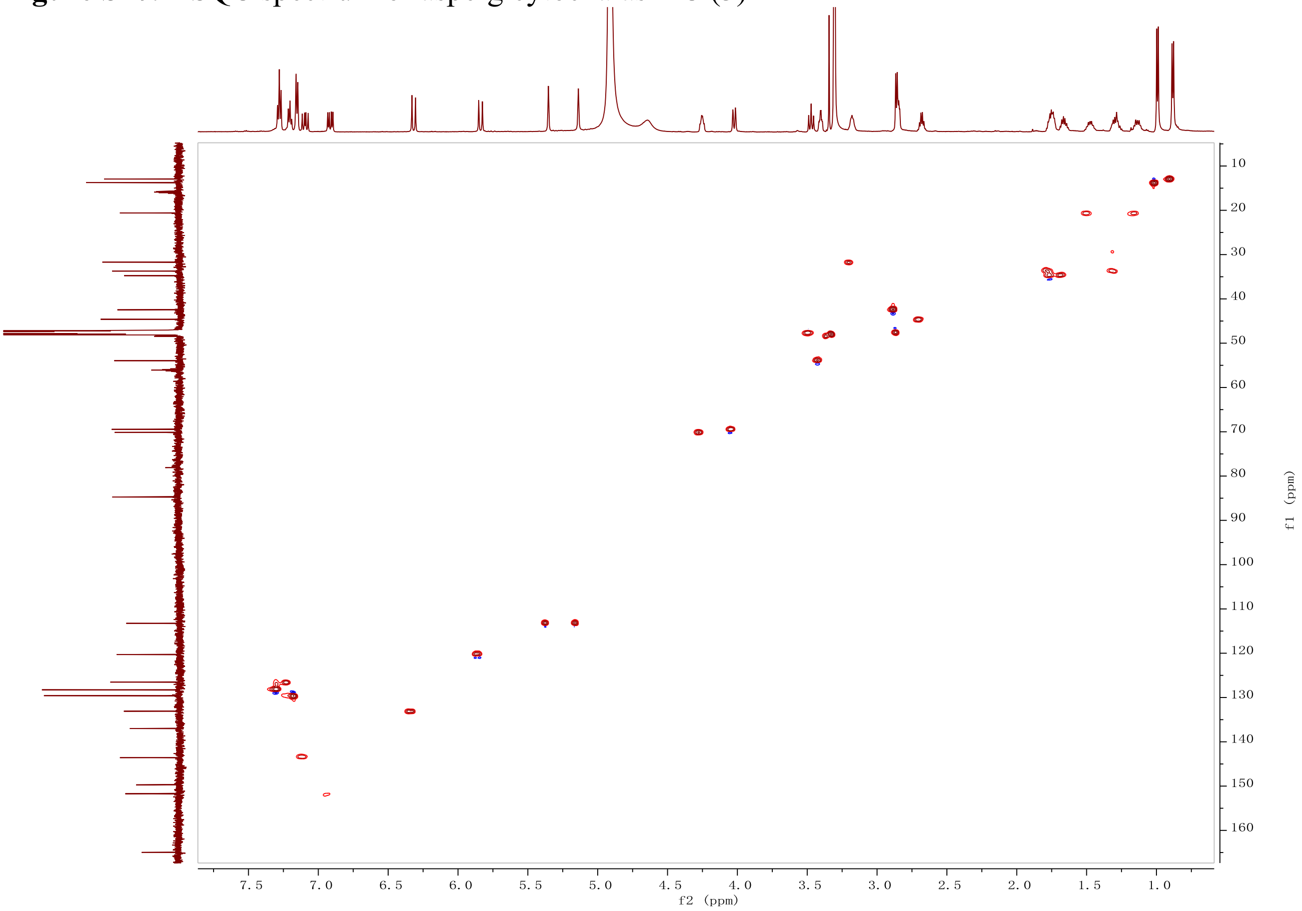

Figure S18. HMBC spectrum of aspergicytochalasin C (**3**)

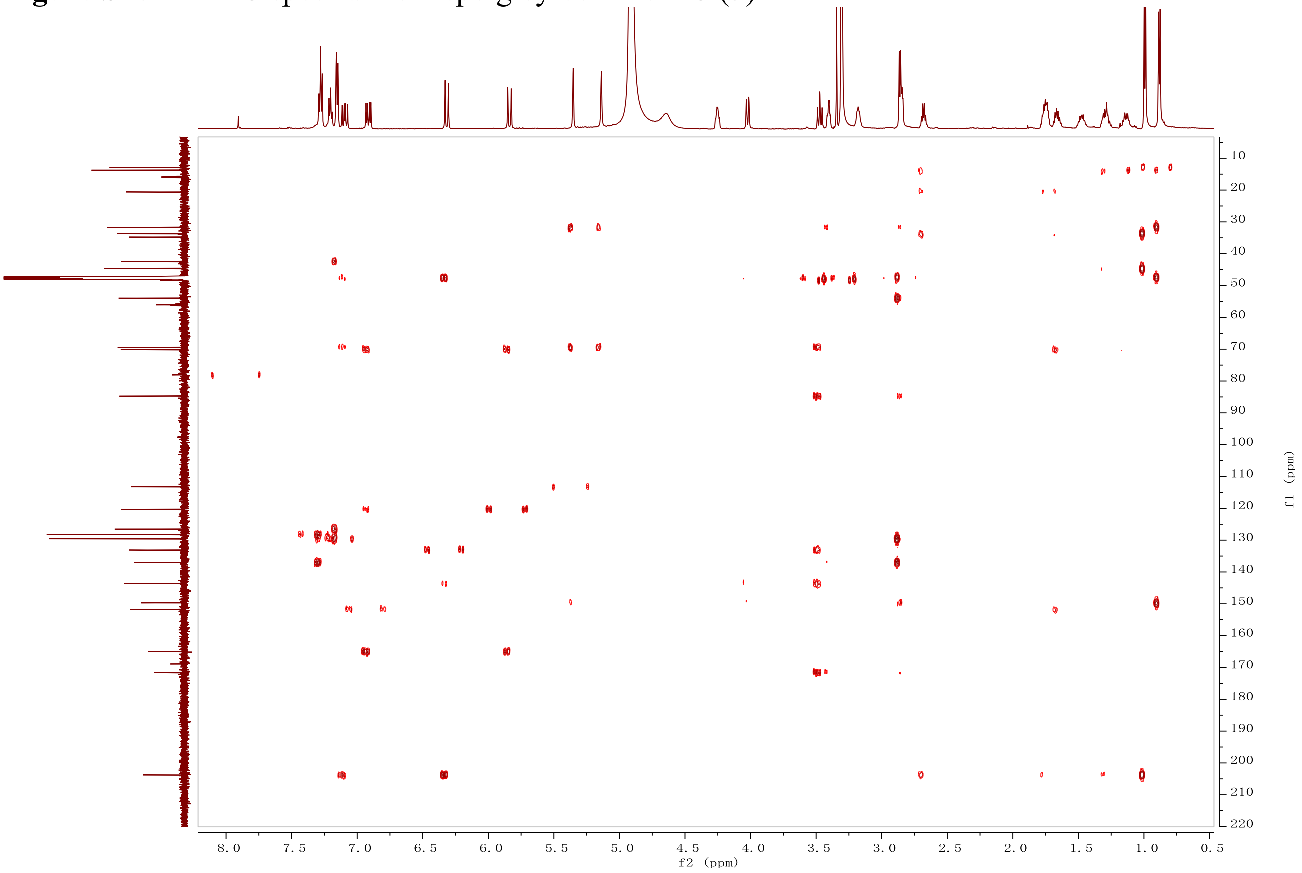

Figure S19. ^{1}H-^{1}H COSY spectrum of aspergicytochalasin C (**3**)

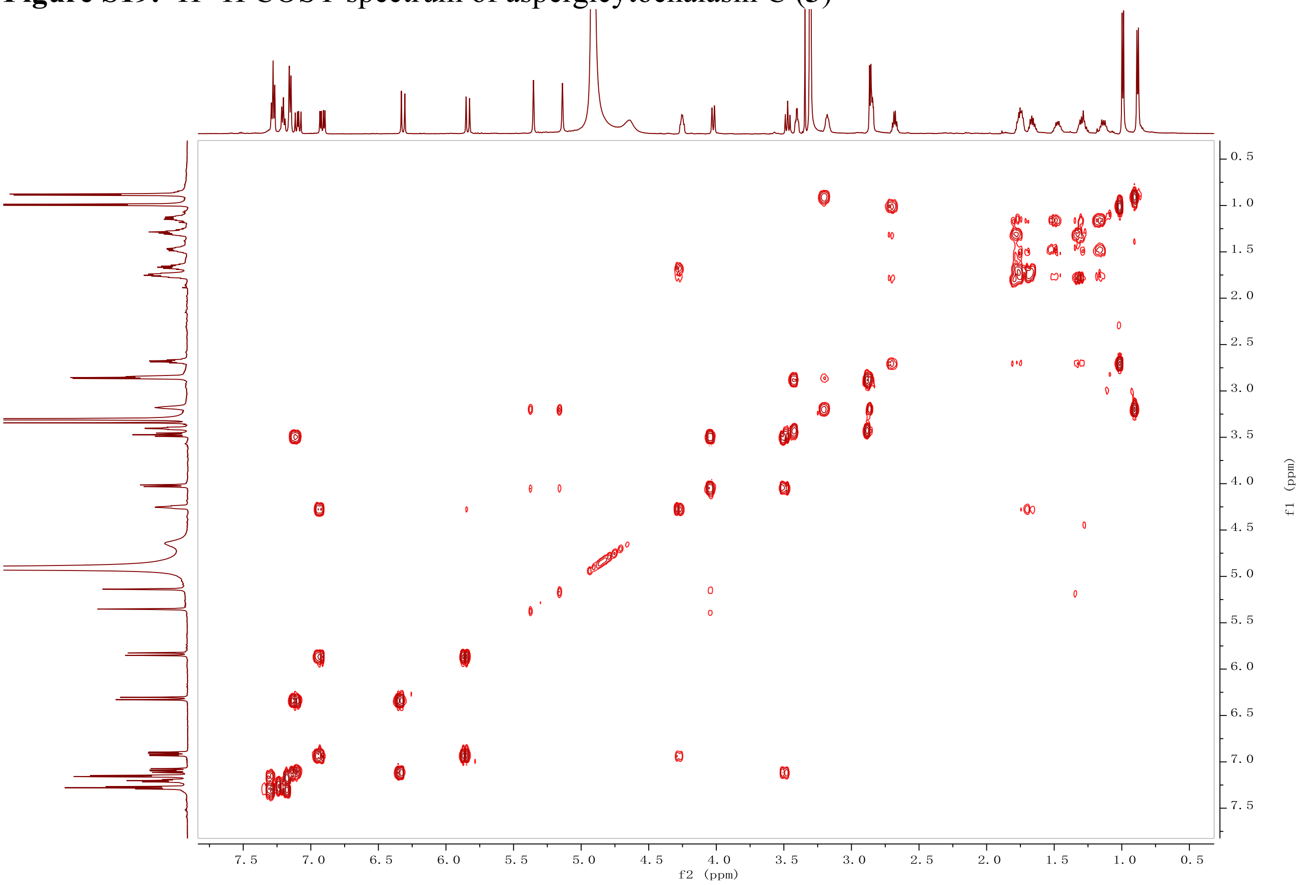

Figure S20. ROESY spectrum of aspergicytochalasin C (**3**)

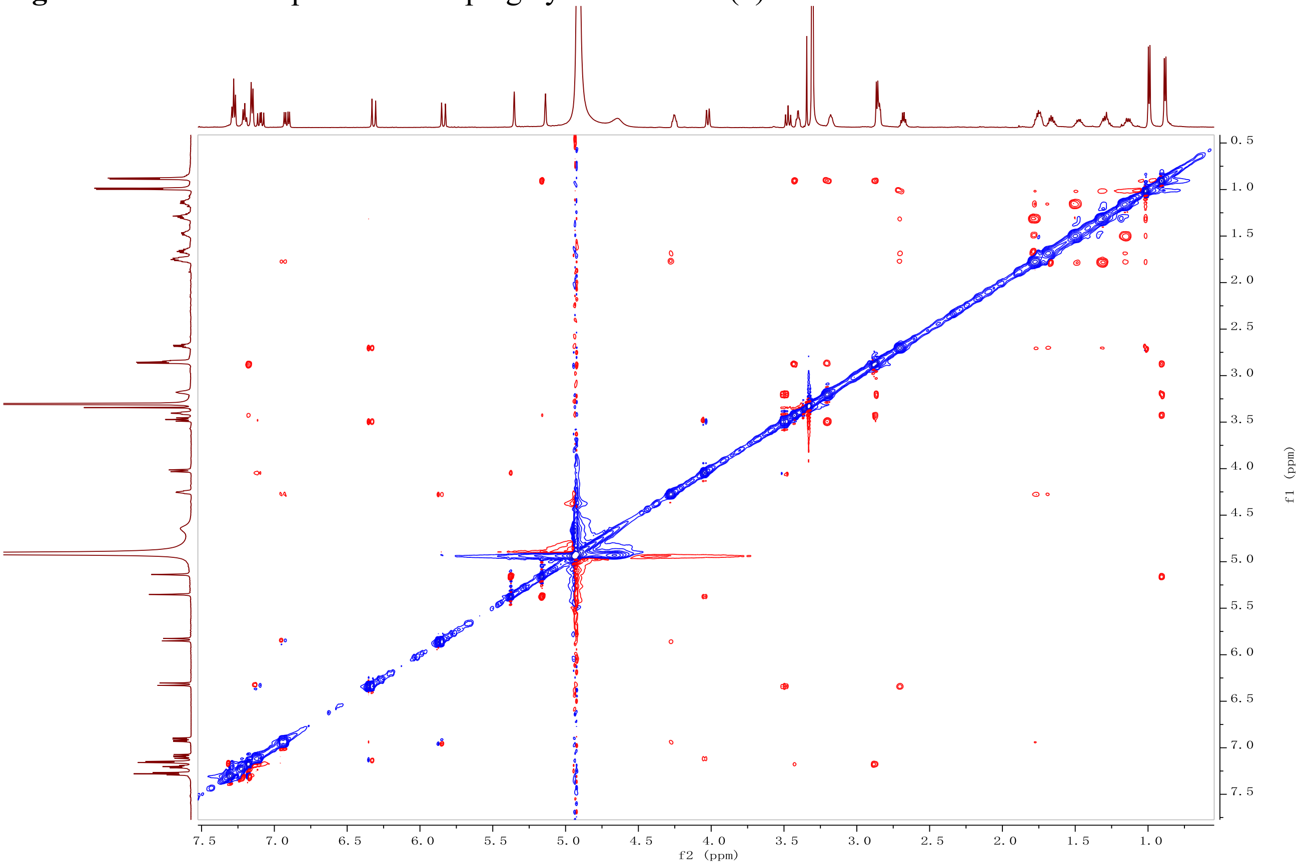

Figure S21. HRESIMS of aspergicytochalasin C (**3**)

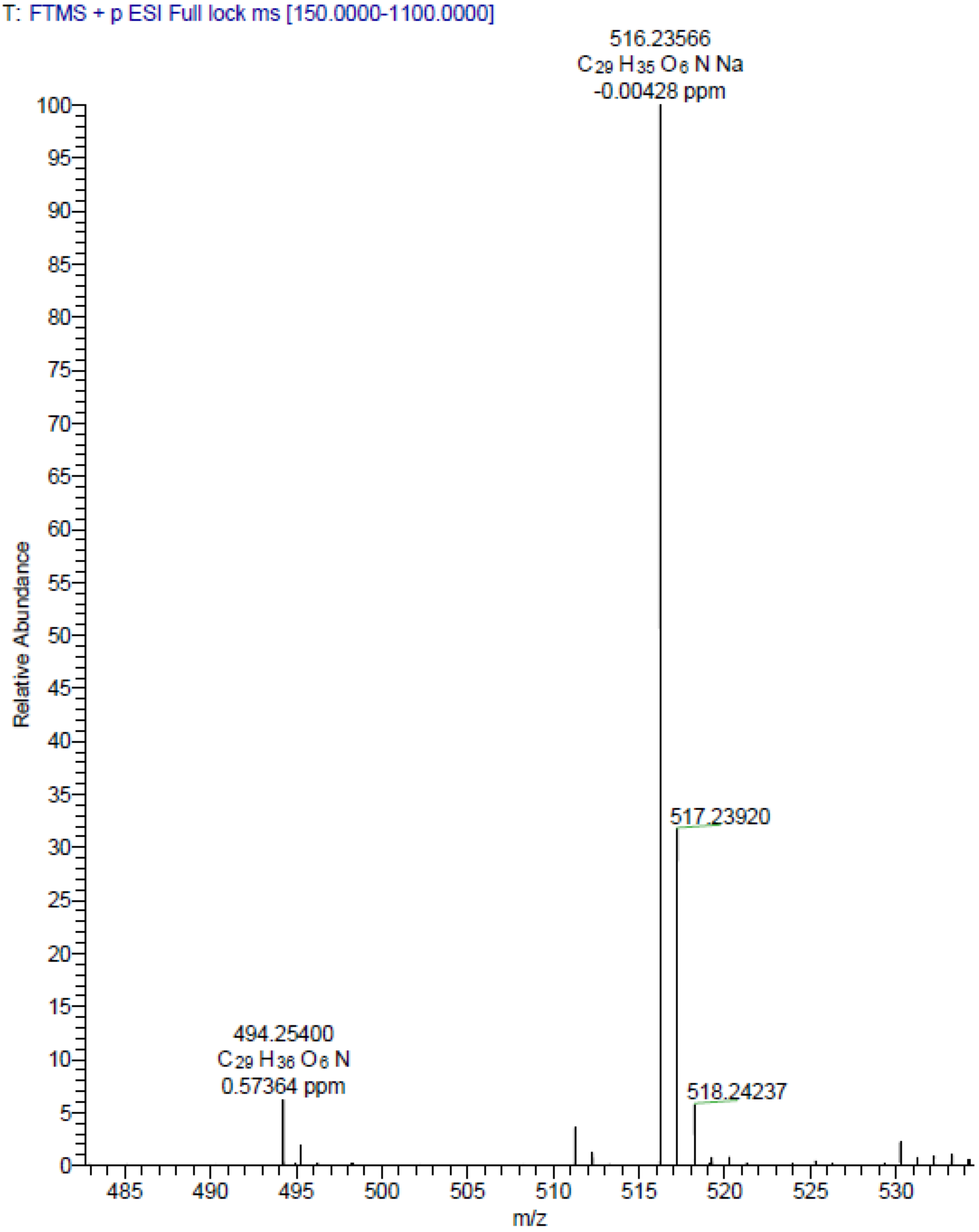

Figure S22. ^{1}H NMR (600 MHz, methanol-d_4) spectrum of aspergicytochalasin D (**4**)

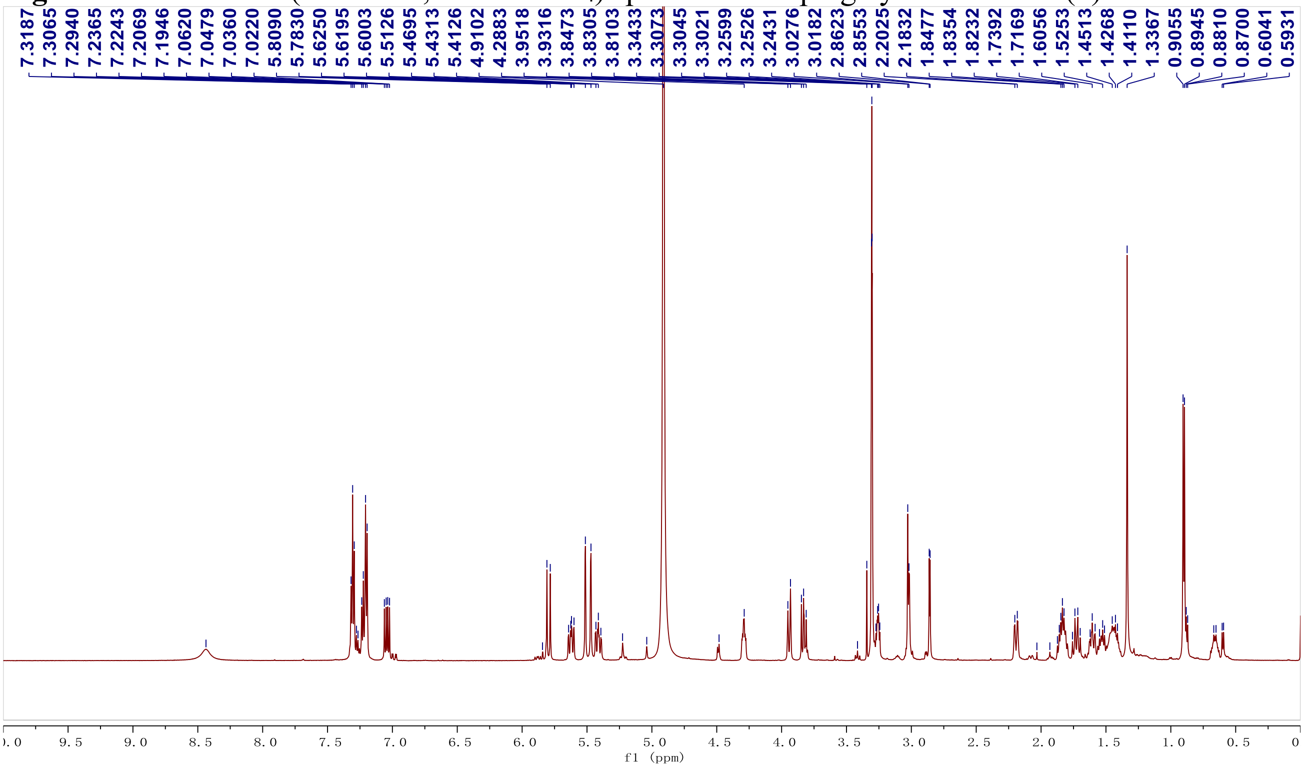

Figure S23. ^{13}C NMR (150 MHz, methanol-d_4) spectrum of aspergicytochalasin D (**4**)

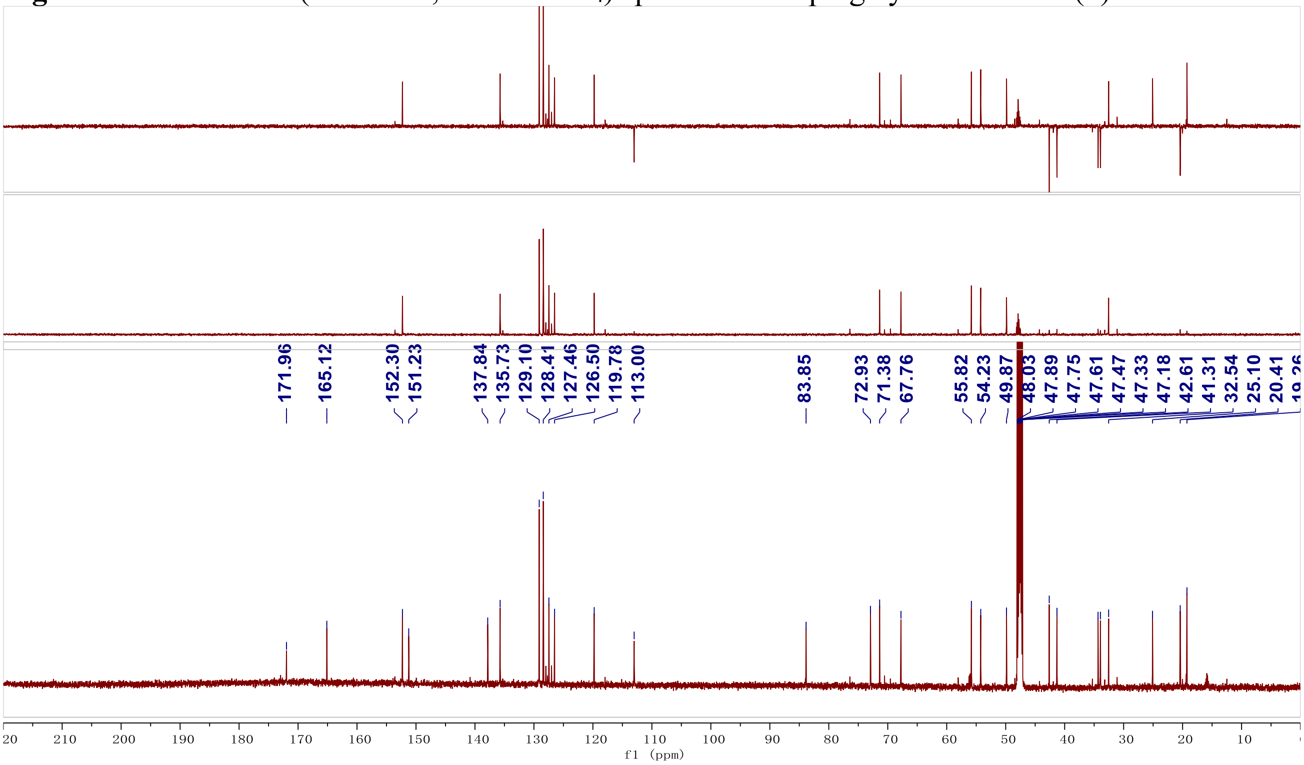

Figure S24. HSQC spectrum of aspergicytochalasin D (**4**)

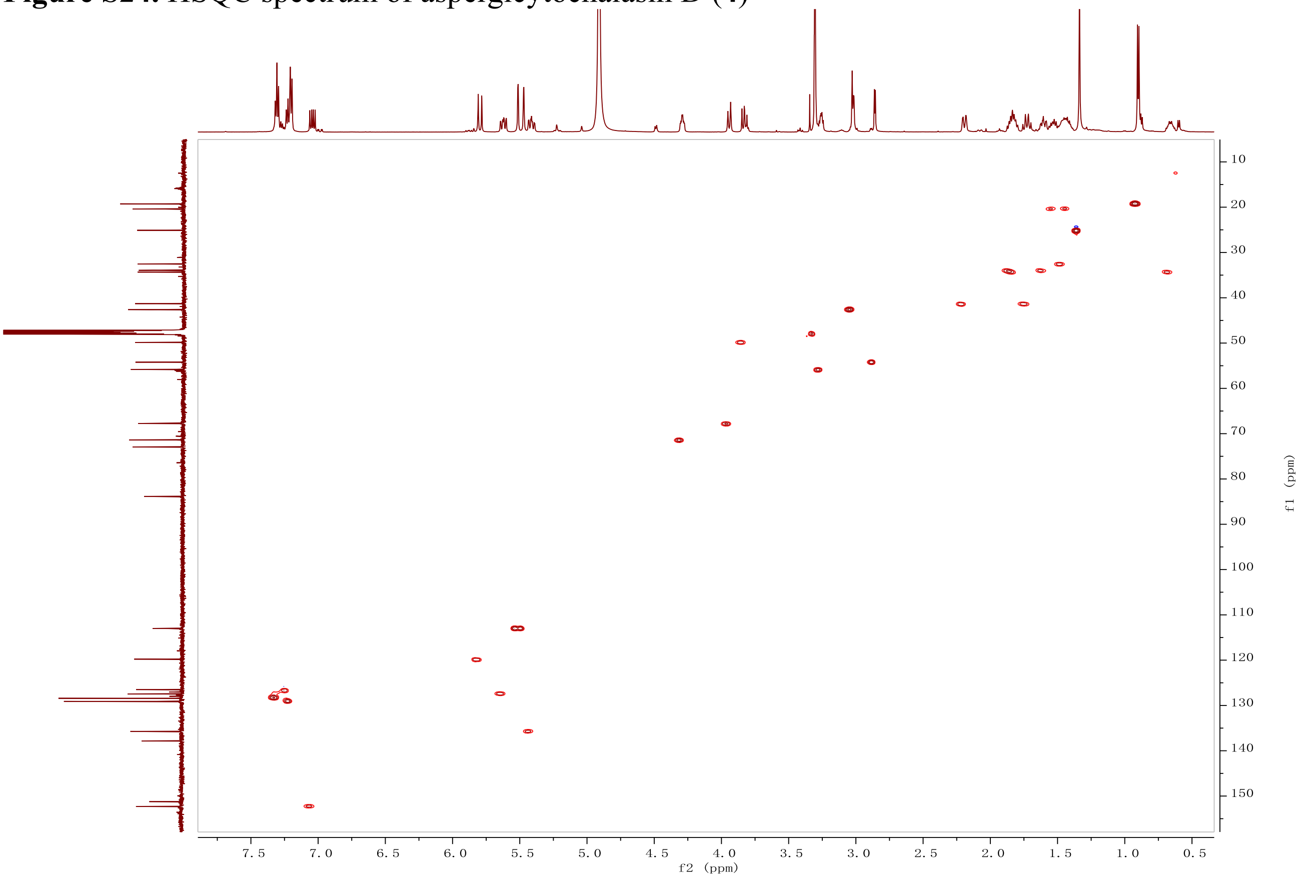

Figure S25. HMBC spectrum of aspergicytochalasin D (**4**)

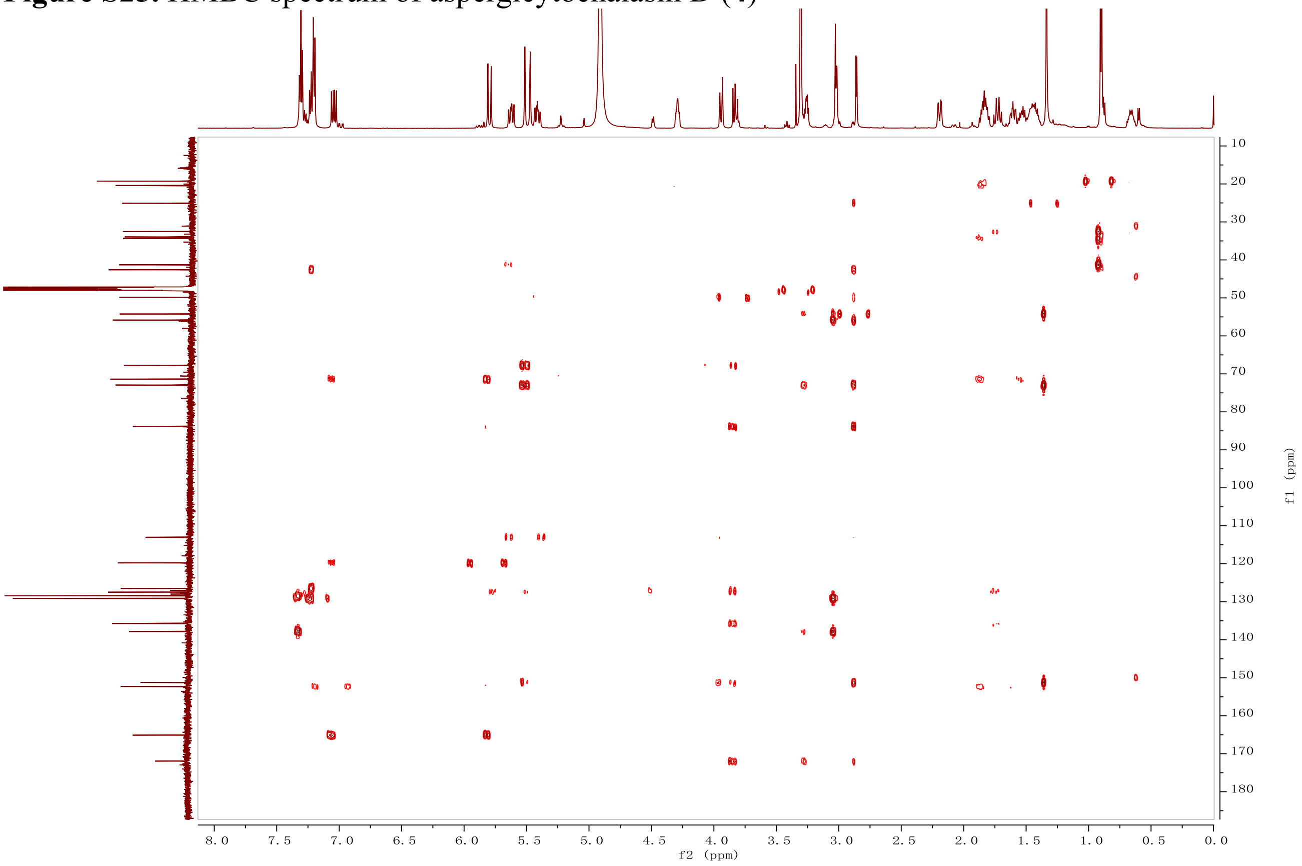

Figure S26. ^{1}H-^{1}H COSY spectrum of aspergicytochalasin D (**4**)

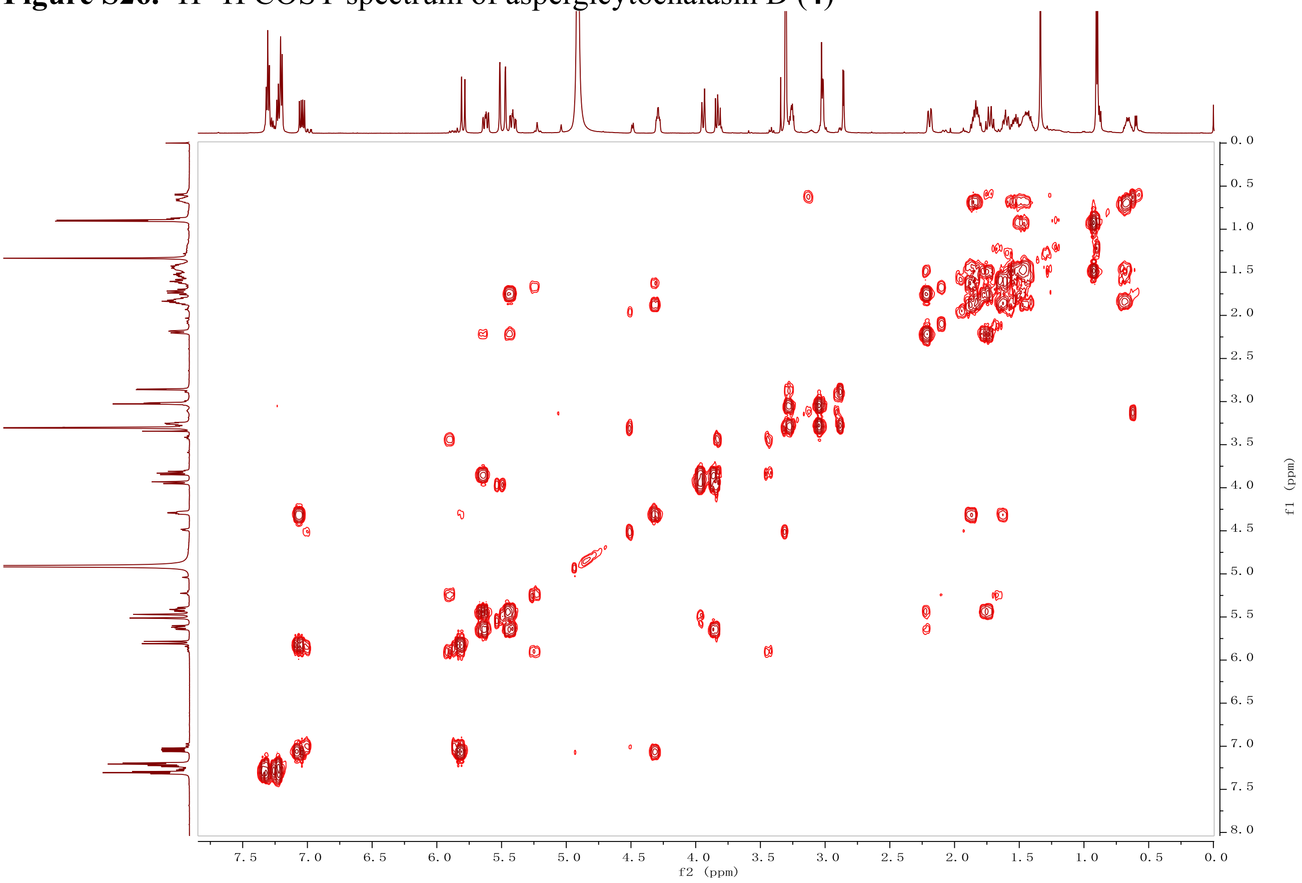

Figure S27. ROESY spectrum of aspergicytochalasin D (**4**)

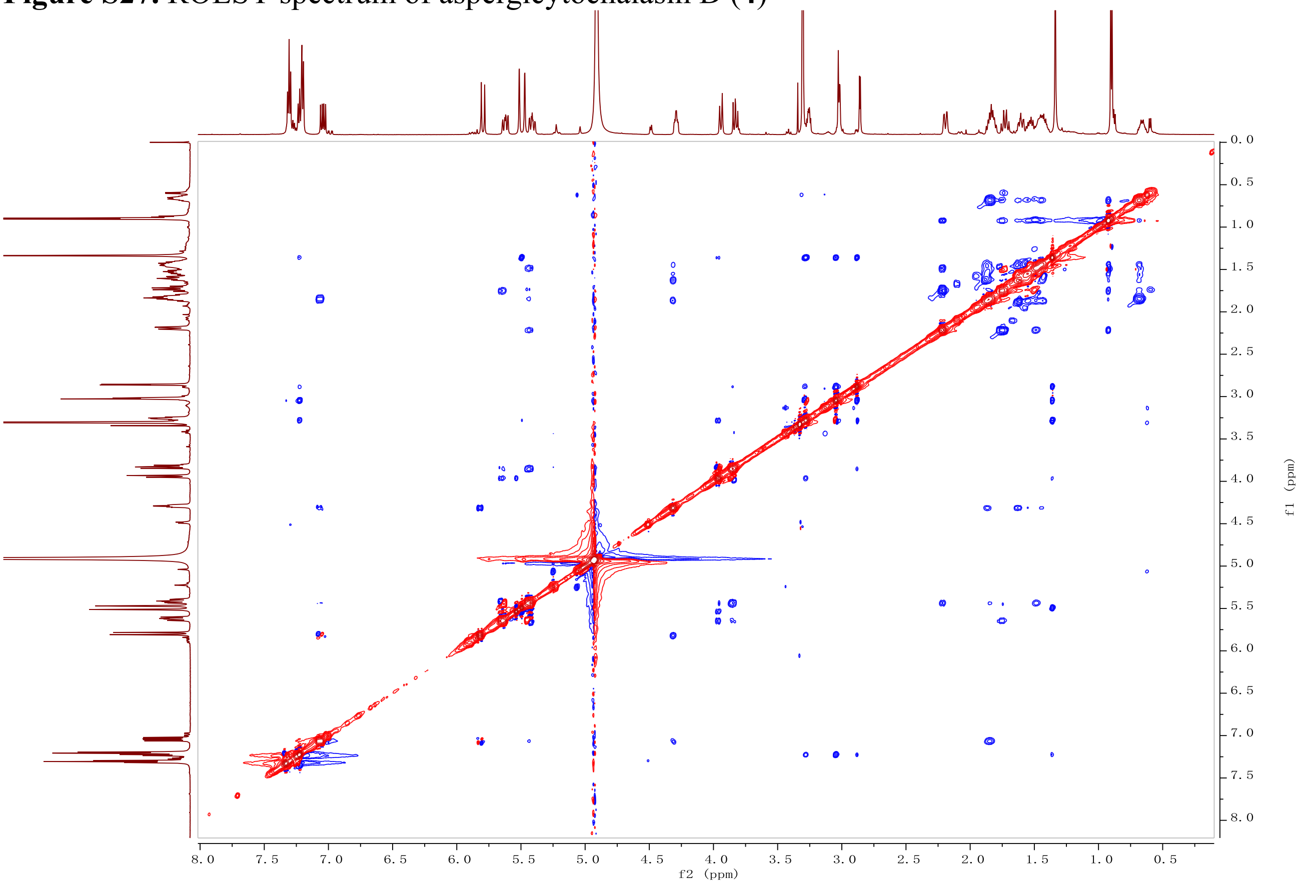

Figure S28. HRESIMS of aspergicytochalasin D (**4**)

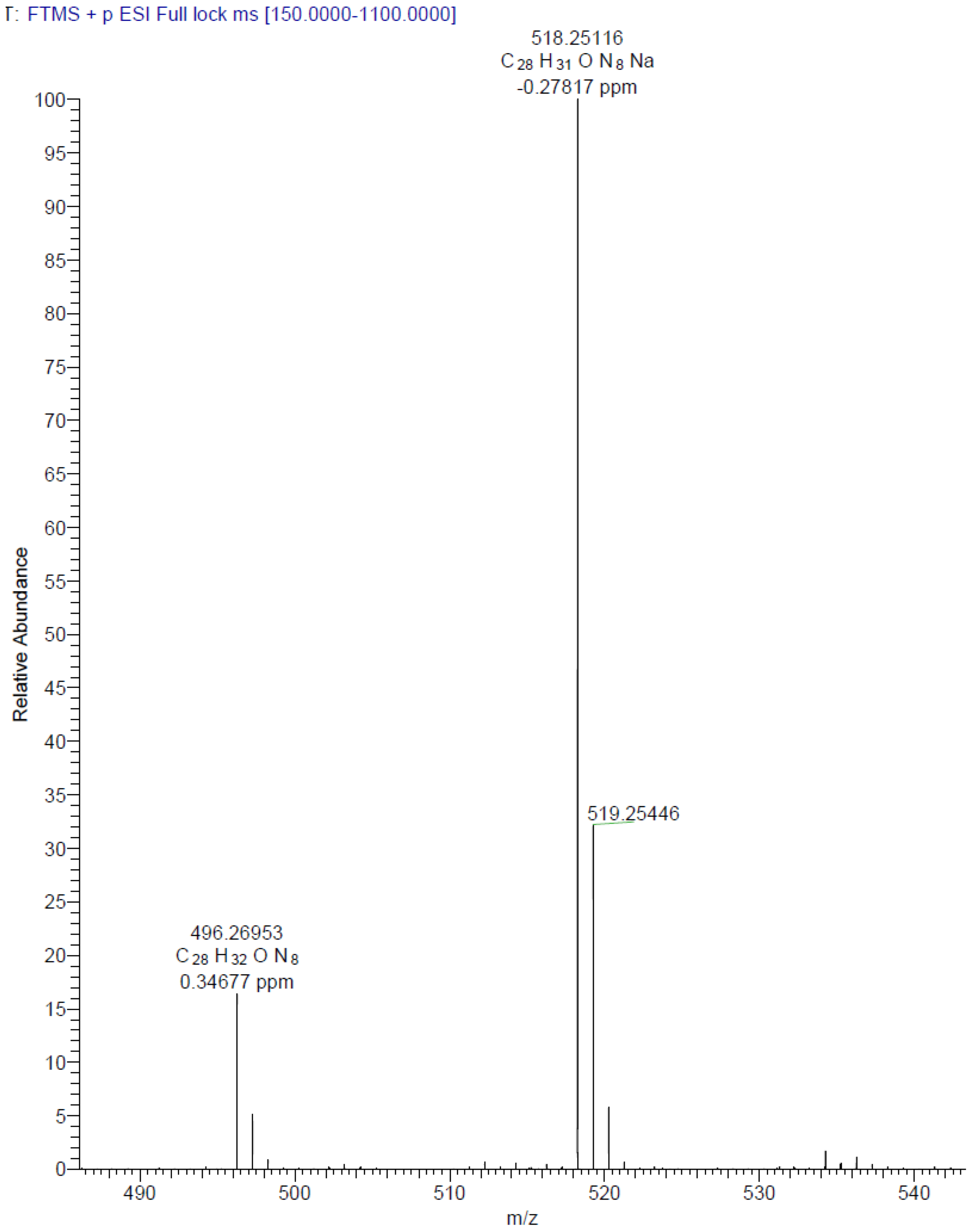

Figure S29. ^{1}H NMR (600 MHz, $CDCl_3$) spectrum of aspergicytochalasin E (**5**)

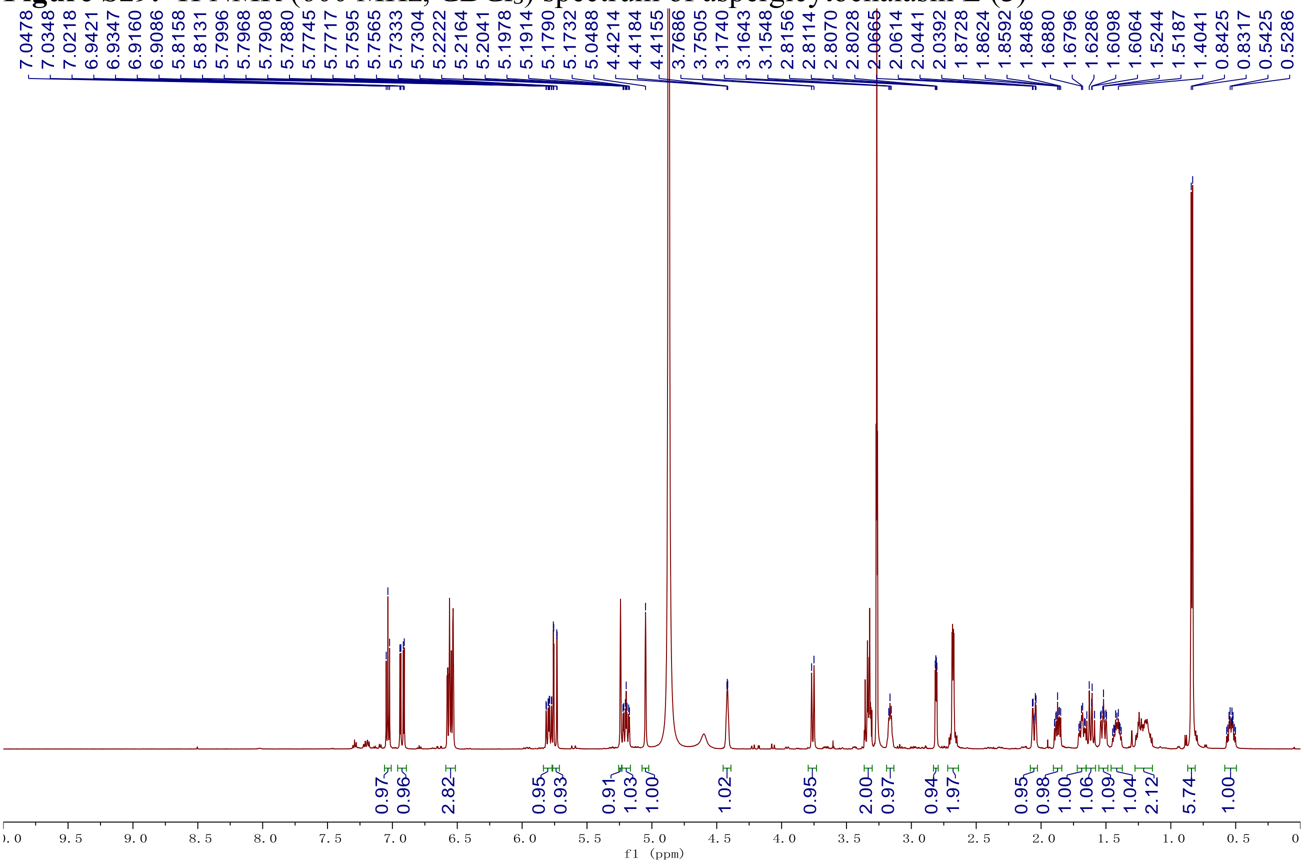

Figure S30. ^{13}C NMR (150 MHz, $CDCl_3$) spectrum of aspergicytochalasin E (**5**)

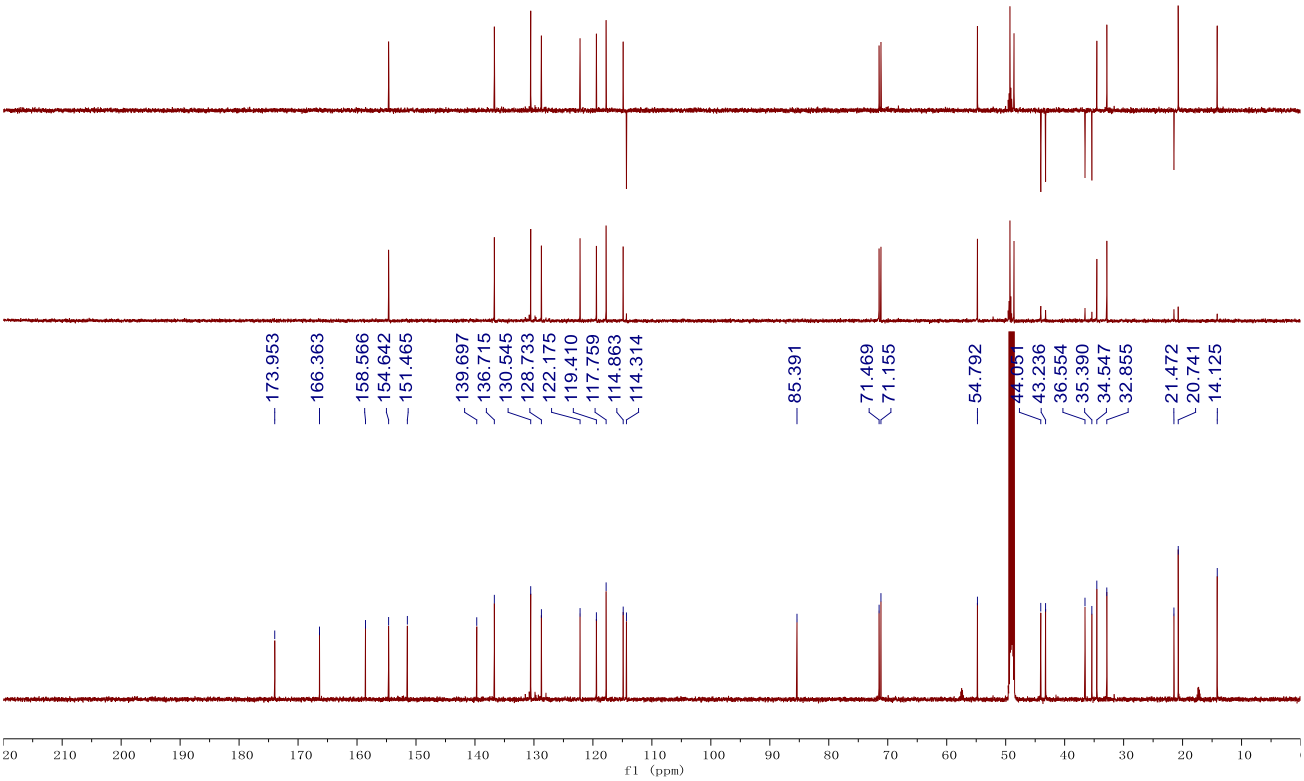

Figure S31. HSQC spectrum of aspergicytochalasin E (**5**)

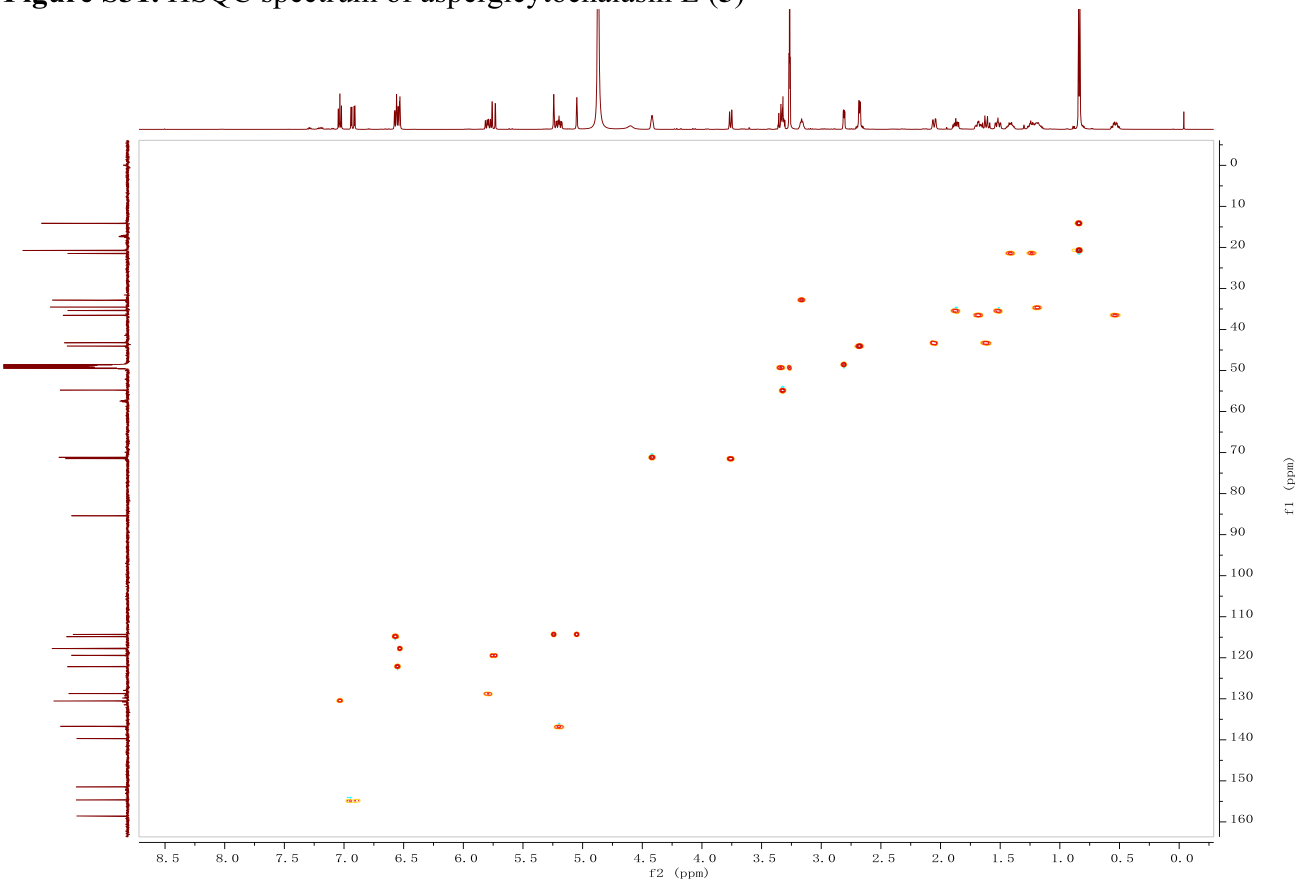

Figure S32. HMBC spectrum of aspergicytochalasin E (**5**)

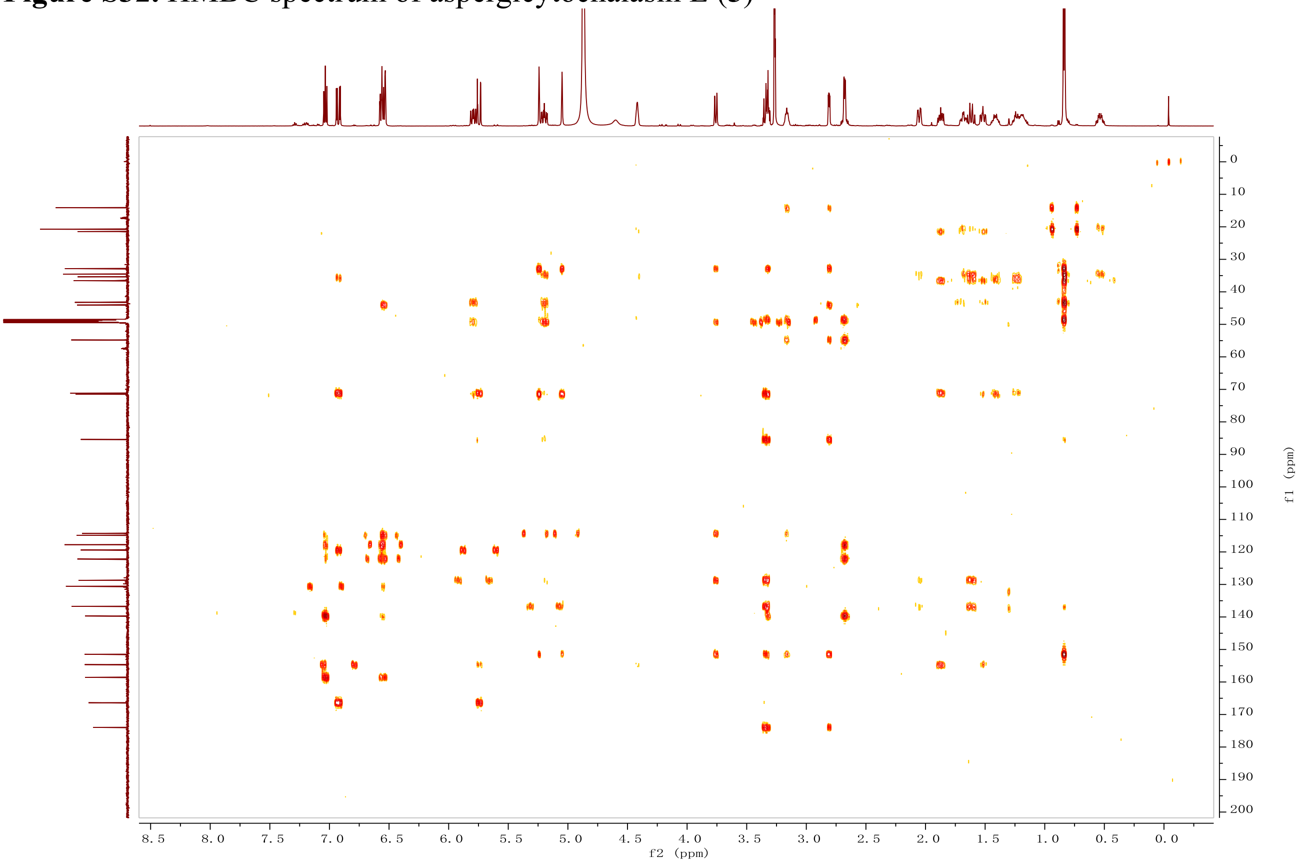

Figure S33. ^{1}H-^{1}H COSY spectrum of aspergicytochalasin E (**5**)

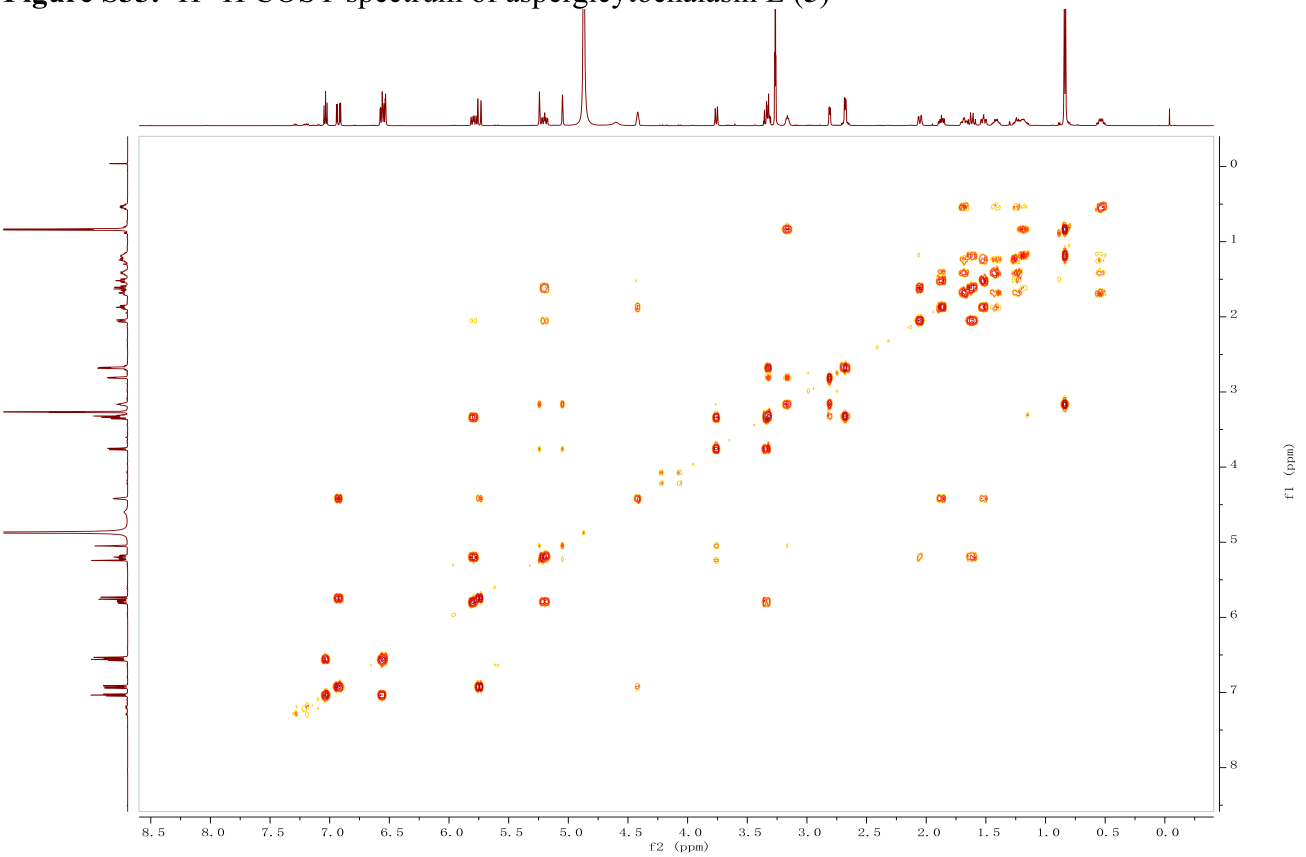

Figure S34. ROESY spectrum of aspergicytochalasin E (**5**)

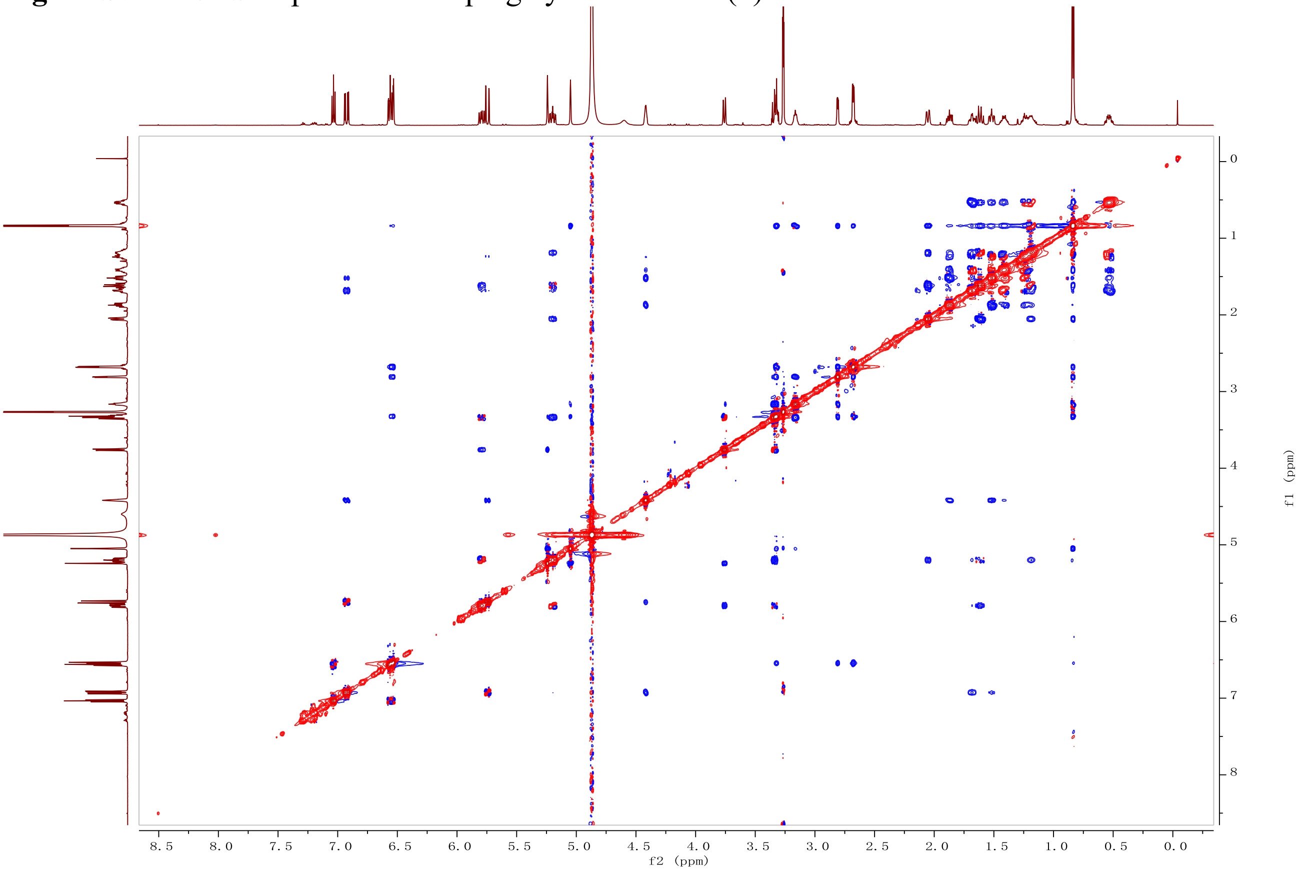

Figure S35. HRESIMS of aspergicytochalasin E (**5**)

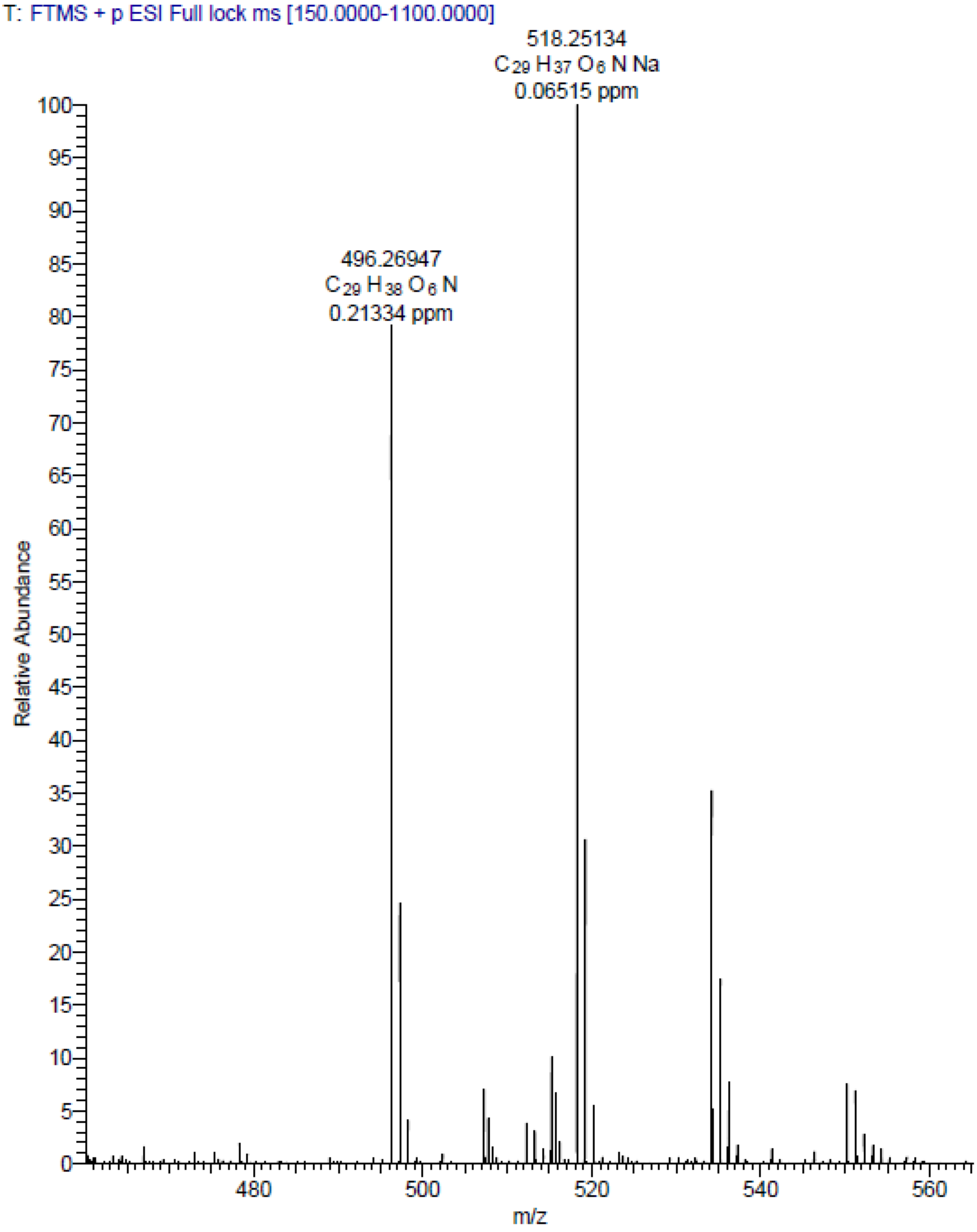

Figure S36. ^{1}H NMR (600 MHz, methanol-d_4) spectrum of aspergicytochalasin F (**6**)

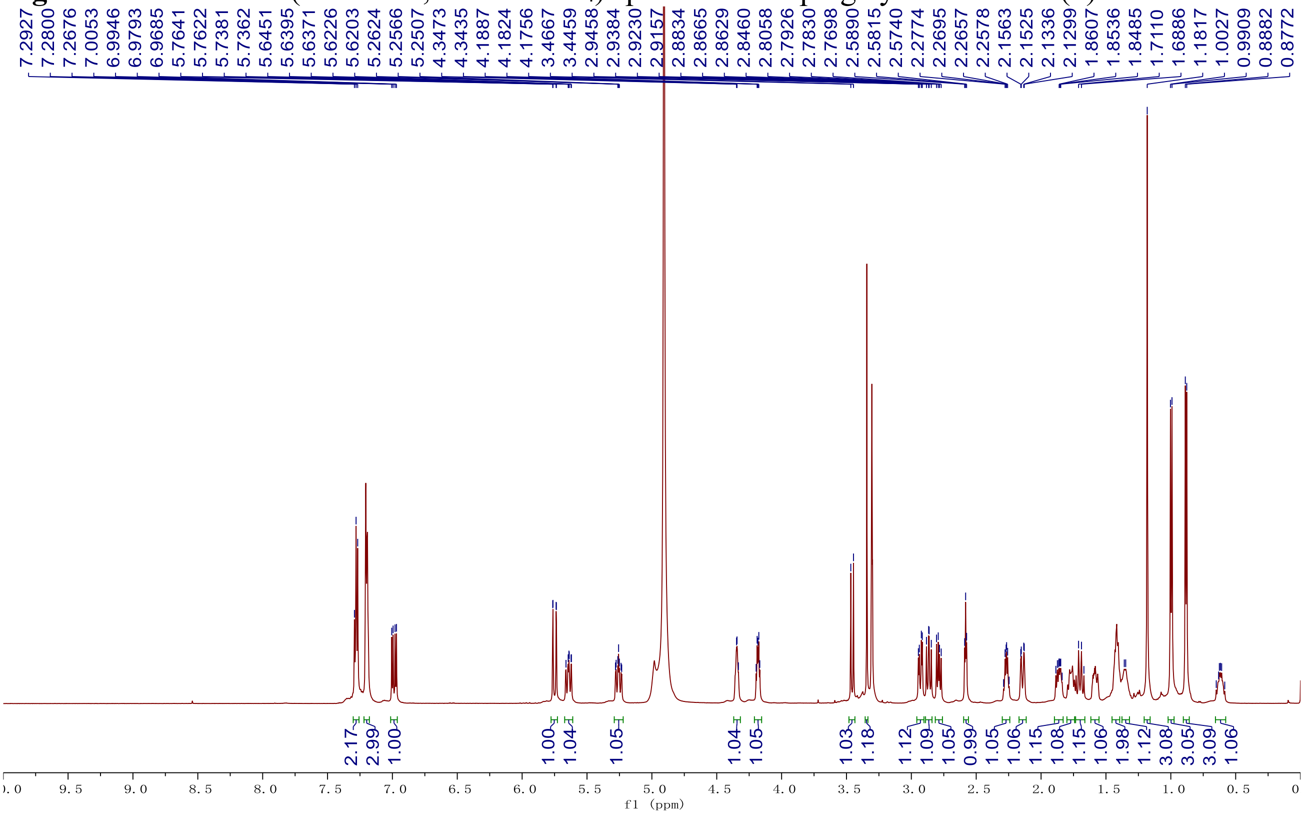

Figure S37. ^{13}C NMR (150 MHz, methanol-d_4) spectrum of aspergicytochalasin F (**6**)

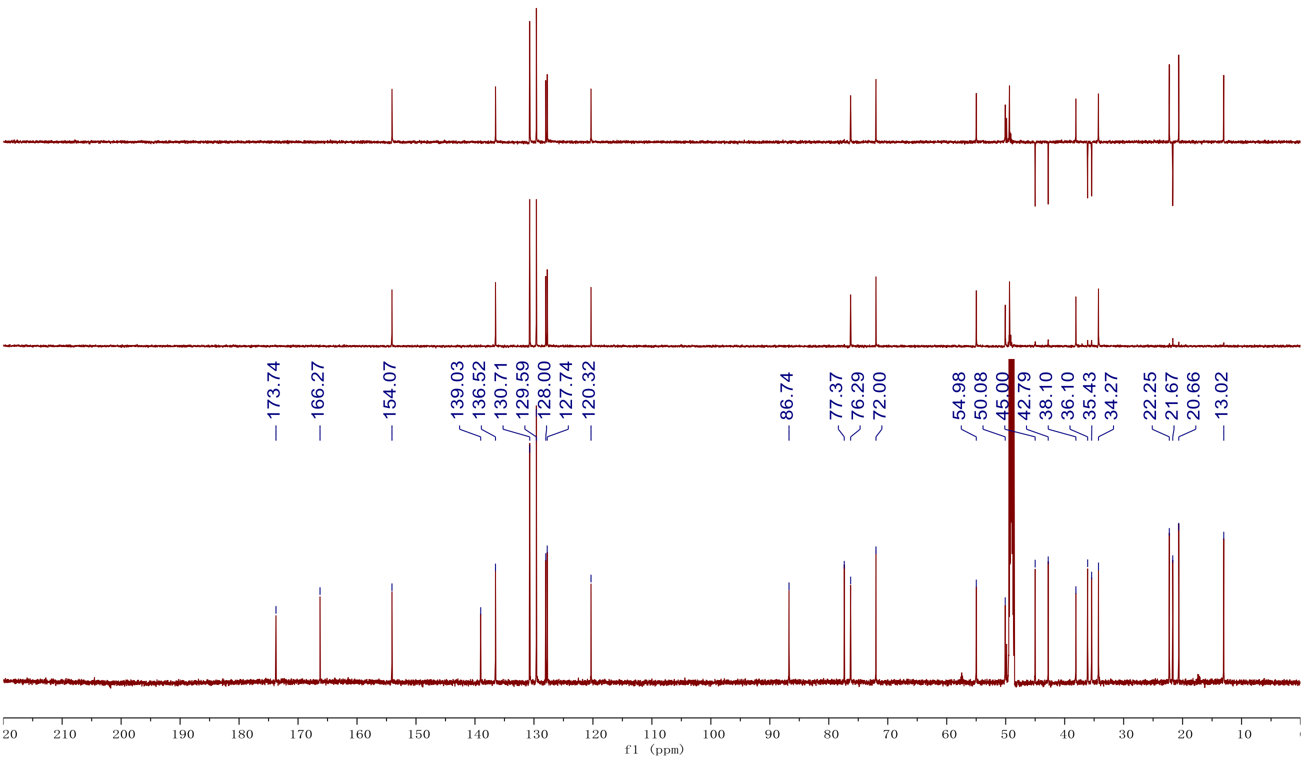

Figure S38. HSQC spectrum of aspergicytochalasin F (**6**)

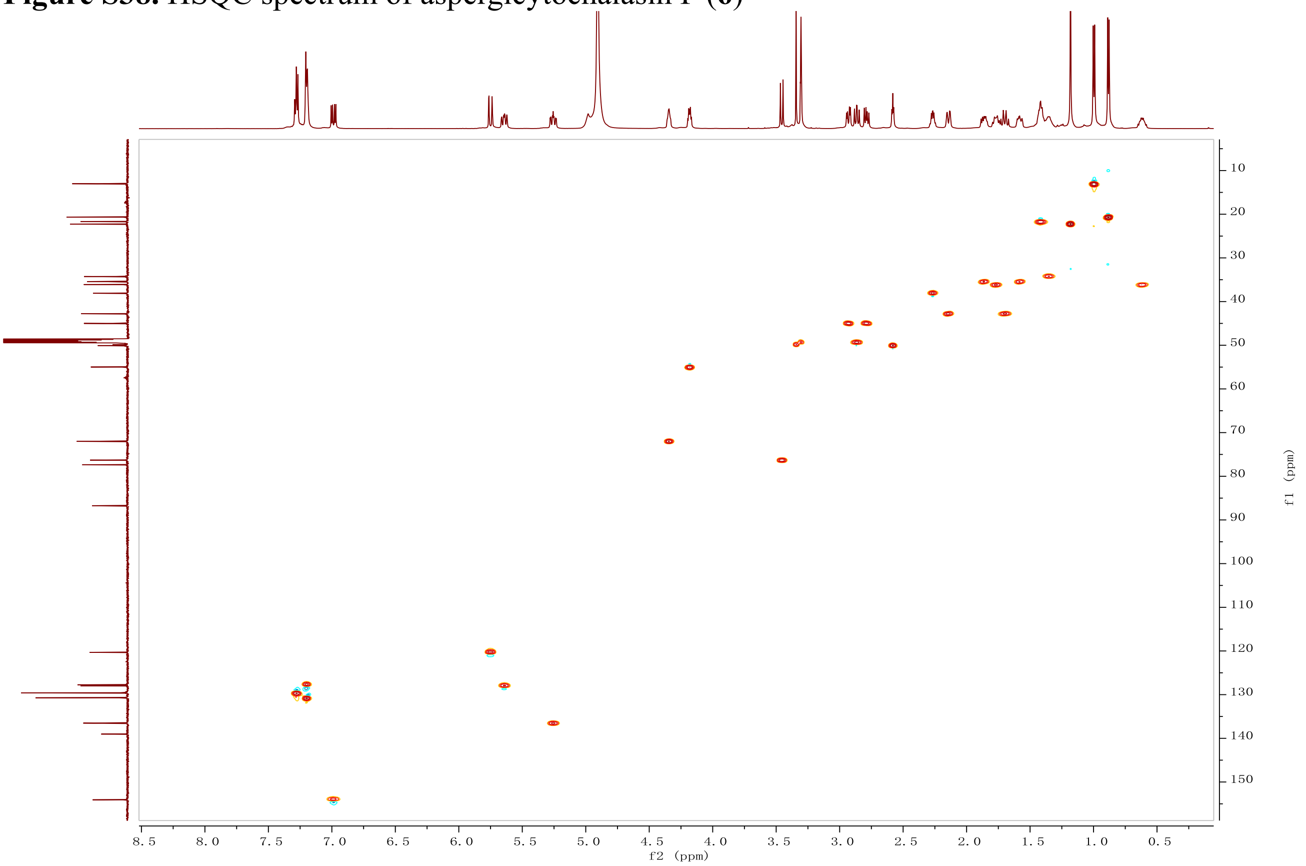

Figure S39. HMBC spectrum of aspergicytochalasin F (**6**)

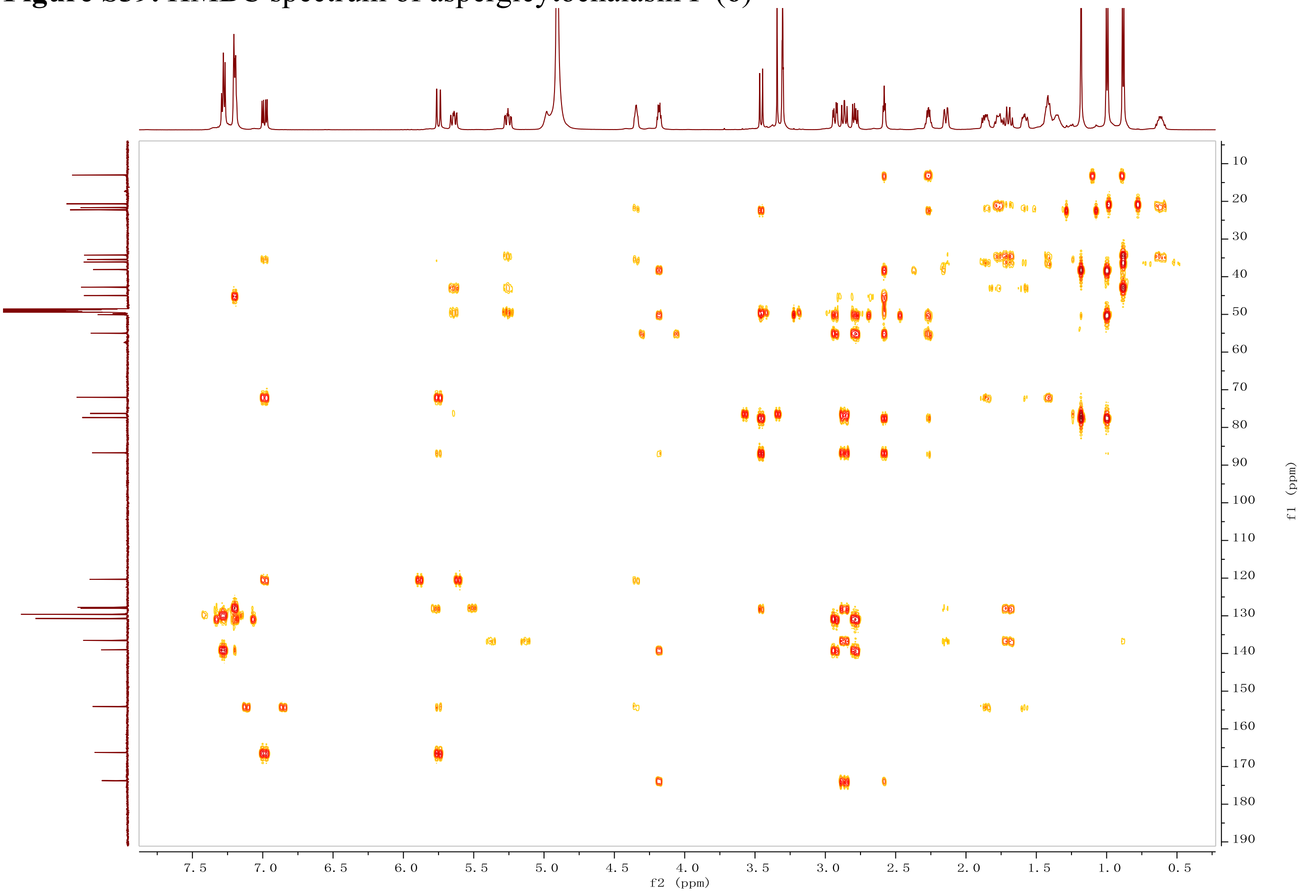

Figure S40. ^{1}H-^{1}H COSY spectrum of aspergicytochalasin F (**6**)

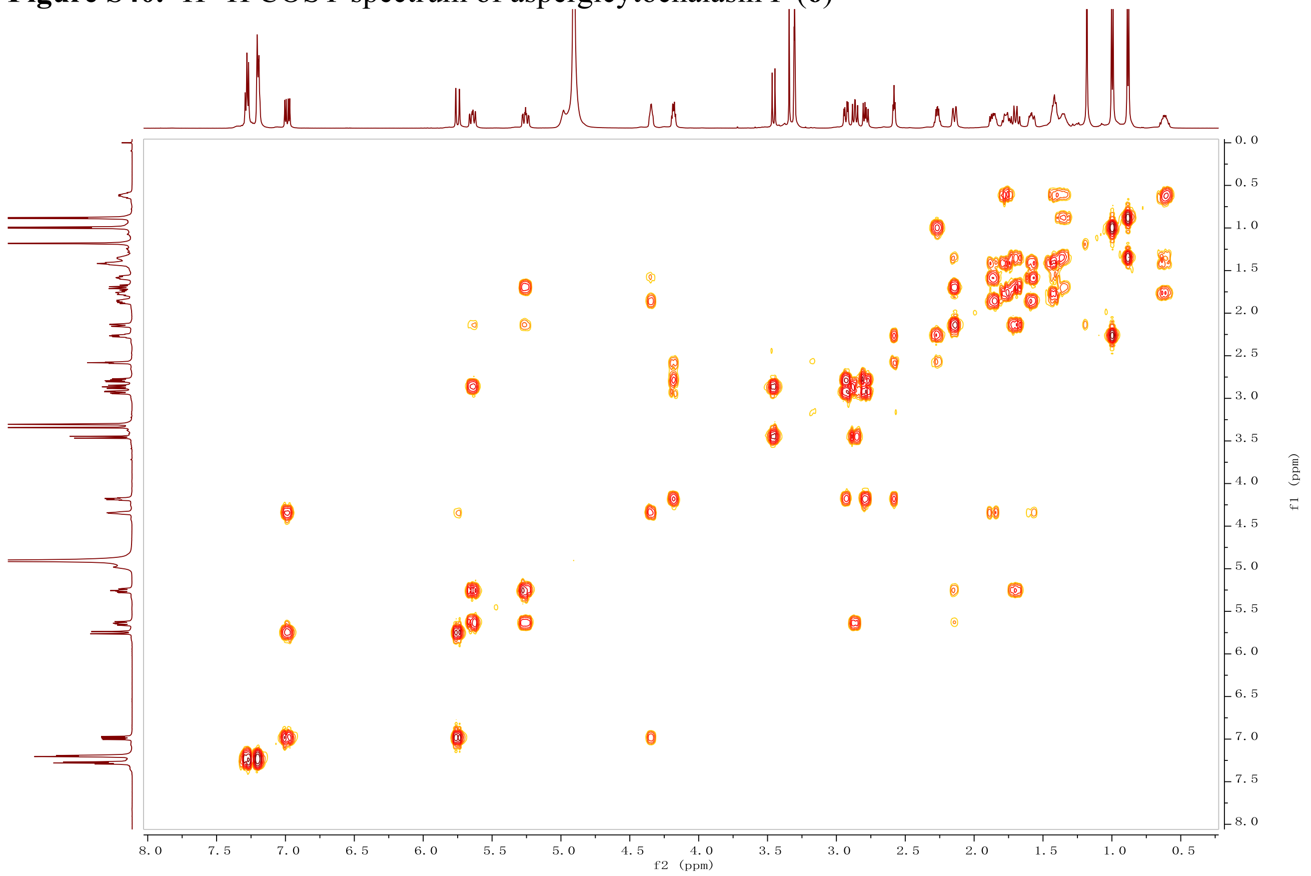

Figure S41. ROESY spectrum of aspergicytochalasin F (**6**)

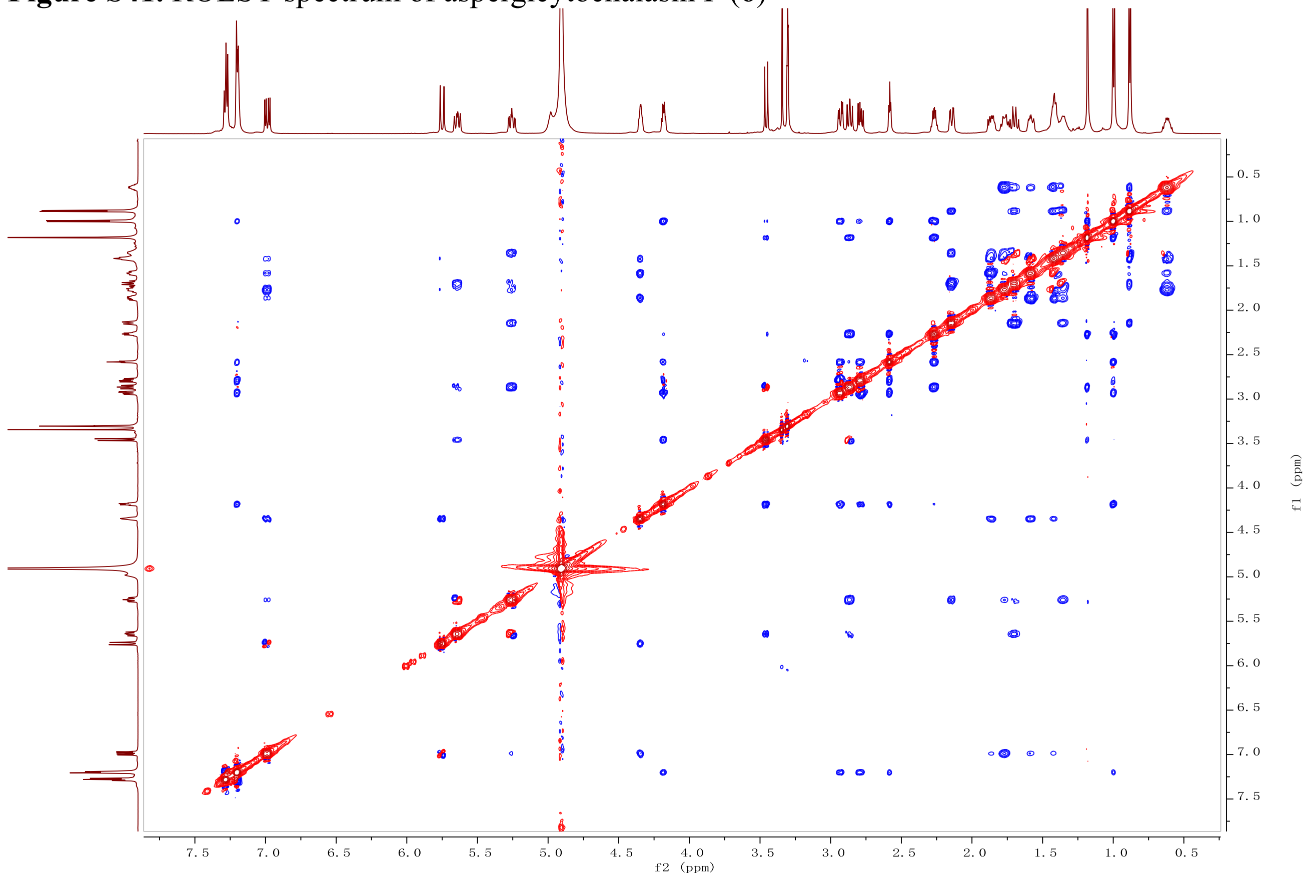

Figure S42. HRESIMS of aspergicytochalasin F (**6**)

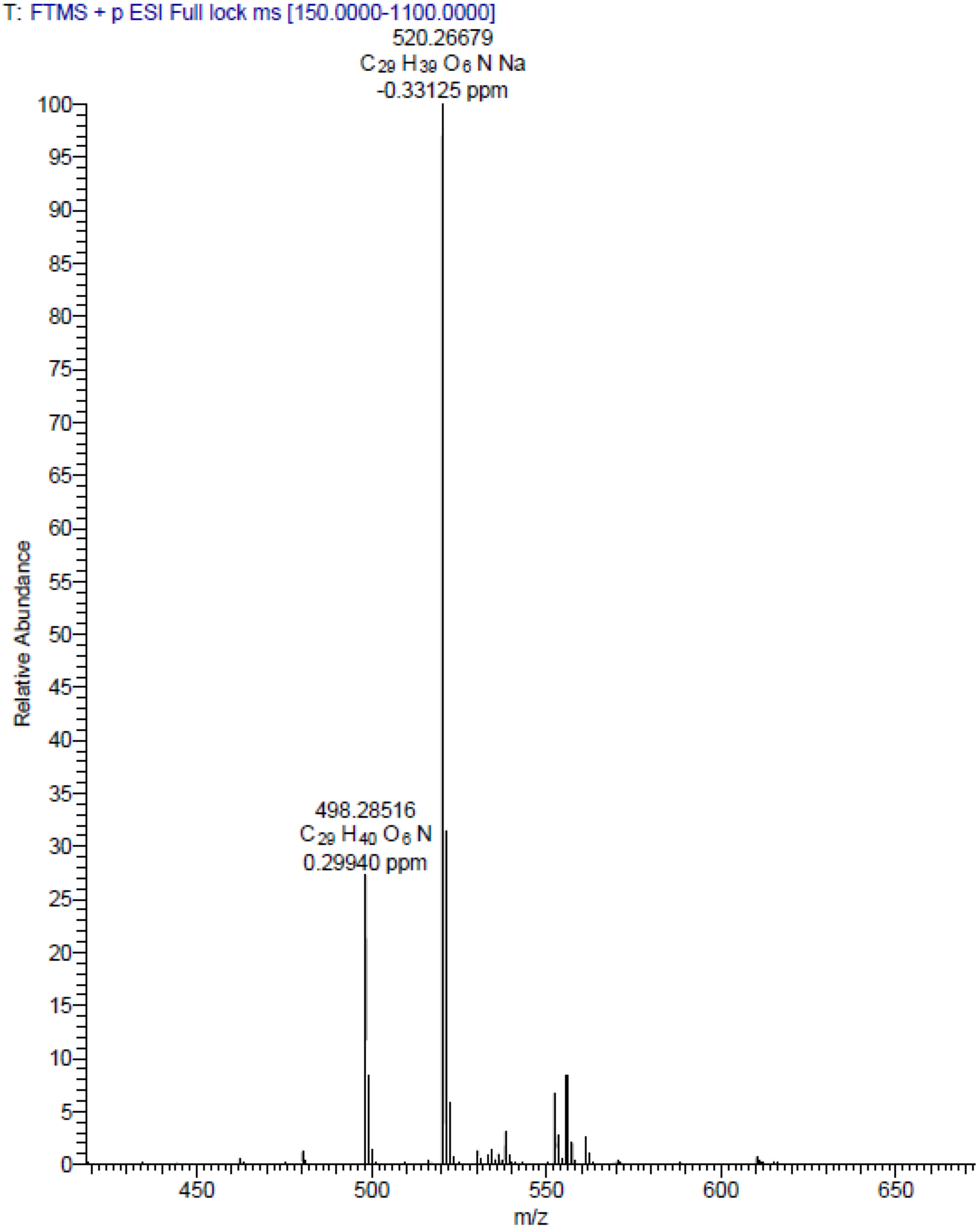

Section S2. X-Ray Crystallographic Data for aspergicytochalasin A (1).

Crystal data for cu_**1**_0m: A light colorless PRISM-like of $C_{29}H_{37}NO_5 \bullet H_2O$, M = 497.61, approximate dimensions 0.128 mm x 0.131 mm x 0.262 mm, was used for the X-ray crystallographic analysis on the BRUKER D8 QUEST. The integration of the data using a monoclinic unit cell yielded a total of 33558 reflections to a maximum θ angle of 79.22° (0.78 Å resolution), of which 5713 were independent (average redundancy 5.874, completeness = 99.7%, R_{int} = 5.23%, R_{sig} = 3.83%) and 5592 (97.88%) were greater than $2\sigma(F^2)$. The final cell constants of a = 9.8035(6) Å, b = 12.4869(8) Å, c = 11.0745(7) Å, α = 90.00°, β = 98.951(2)°, γ = 90.00°, V = 1339.18(15) Å^3, T = 150.(2) K. Data were corrected for absorption effects using the Multi-Scan method (SADABS). The structure was solved and refined using the Bruker SHELXTL Software Package, using the space group P 1 21 1, with Z = 2, μ(CuKα) = 1.54178. The final anisotropic full-matrix least-squares refinement on F^2 with 344 variables converged at R1 = 3.24%, for the observed data and wR2 = 7.65% for all data. The goodness-of-fit was 1.054. The absolute configuration was determined by the Flack parameter = 0.08(4), which was determined using 2534 quotients [(I+)-(I-)]/[(I+)+(I-)](Parsons, Flack and Wagner, Acta Cryst. B69 (2013) 249-259).

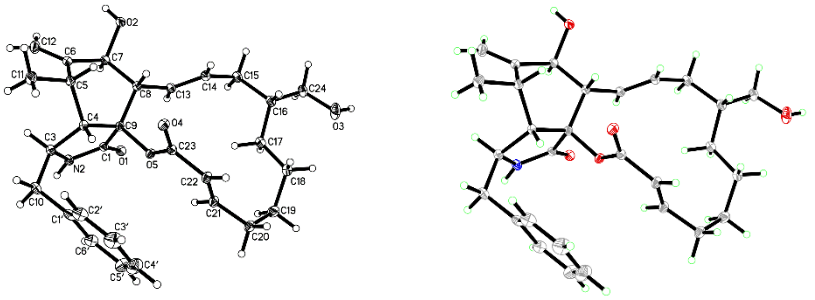

View of a molecule of **1** with the atom-labelling scheme.

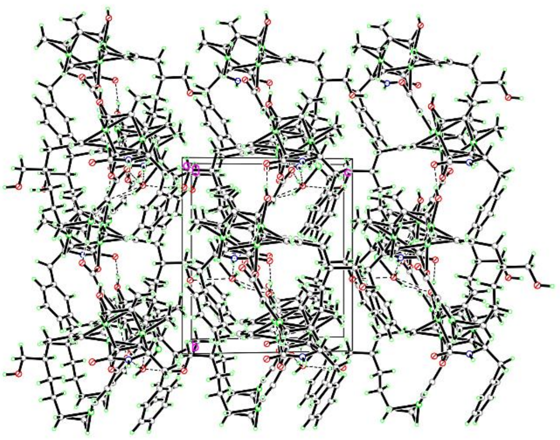

Part of packing of **1**, viewed down the a direction. Dashed lines indicated hydroxy bonds.
